# CD4+FoxP3+ T regulatory cells subsets release small extracellular vesicles containing cell death-related proteins as potential mechanism of T cell suppression

**DOI:** 10.3389/fimmu.2026.1777669

**Published:** 2026-04-21

**Authors:** María José Moya-Guzmán, Javiera de Solminihac, Tomás Carrasco-Loncharic, Thilo Kaehne, Karina Pino-Lagos

**Affiliations:** 1 Facultad de Medicina, Centro de Investigación e Innovación Biomédica, Universidad de los Andes, Santiago, Chile; 2 Institute of Experimental Medicine, Medical Faculty, Otto von Guericke University, Magdeburg, Germany

**Keywords:** apoptosis, exosomes, extracellular vesicles, immune regulation, Tregs, cell death

## Abstract

**Introduction:**

T regulatory cells (Tregs) are pivotal for immune tolerance. Among the suppression mechanisms attributed to Tregs, the release of small extracellular vesicles (sEV) has been proposed with CD73, Nrp1 and the transfer of key miRNAs playing a key role. Although these findings have been described for natural Tregs (nTregs), little is known on Tregs induced *in vitro* (iTregs).

**Methods:**

Here, we characterized sEV production from three types of Tregs: nTregs and *in vitro* iTregs produced with TGF-β or with TGF-β plus retinoic acid (RA) (RATregs).

**Results:**

Characterization of sEV production indicates that all Tregs produce sEV with similar size and presence of Alix and Tsg101, with RATregs showing the highest production of sEV. Regarding sEV function, nTregs, iTregs and RATregs produce sEV that suppress CD4+ T and CD8+ T cell proliferation. Interestingly, *in vitro* culture of splenocytes with sEV showed induction of cell death/apoptosis, which is enhanced when adding sEV obtained from nTregs and RATregs, and not from iTregs. Proteomic analysis on sEV obtained from Tregs subsets revealed 163 common proteins, of which some share Tregs biology-related functions such as Apolipoprotein AI, Integrin β2, Lactate Dehydrogenase-A, Thrombospondin 1 and Transferrin Receptor. Depending on the combination of Tregs subsets analyzed, 5 major clusters containing cell death-related proteins were identified, including molecules belonging to the Granzyme/Perforin pathway.

**Discussion:**

Although this study does not explain the enhanced cell death observed in cultures with sEV produced by nTregs and RATregs, it does demonstrate that Tregs subsets produce sEV with inhibitory function driven -at least- by cell death, providing new insights on the biology of Tregs.

## Introduction

T regulatory cells (Tregs) are the main immune cell type responsible for generating and maintaining immune tolerance, thus, their function is of great importance under pathogenic circumstances such as autoimmunity, transplant rejection, allergy and other immune diseases (1). Tregs can be classified based on their origin: thymic or natural Tregs (nTregs) corresponding to the cells generated in the thymus, and peripheral or induced Tregs (iTregs) as those differentiated from conventional CD4+ T cells in the presence of low costimulatory signals and anti-inflammatory cytokines (2, 3). iTregs can also be produced *in vitro*, in which case various protocols have been described but the most common refers to the use of IL-2 and TGF-β (4). In 2007, Benson et al. demonstrated that the use of all-*trans*-retinoic acid (ATRA), the metabolically active form of vitamin A, produces Tregs (RATregs) with stable phenotype since they keep high expression of FoxP3 even under inflammatory conditions (5). Tregs can exert their function through different mechanisms, including the release of anti-inflammatory cytokines, expression of co-inhibitory molecules, consumption of ATP or production of adenosine. In addition, it has been reported that Tregs can exert cytotoxicity on target cells through the activation of cell-death programs driven by granzymes and perforin (6) or modulate immunity through the release of small extracellular vesicles (sEV) (7, 8).

Most reports related to sEV production by Tregs use nTregs as cellular source, whereas only one group have reported sEV production by iTregs, and another described the effects of sEV obtained from RATregs. In the study using iTregs-derived vesicles sEV were associated with the prevention of Th17/Tregs imbalance in mice with arthritis (9), whereas RATregs-derived sEV regulate periodontitis immune response in a CD73-dependent manner (10). With respect to sEV´s cargo, it is well known that the protein content varies greatly depending on the cell source (11). Therefore, we hypothesized that sEV obtained from different subsets of Tregs may be composed by different molecules and exert distinct functions. Thus, the main objective of this report was to characterize and compare sEV from nTregs, iTregs, and RATregs, evaluating suppressive activity and sEV protein composition.

Our data indicate that sEV from different types of Tregs share similar characteristics (size and sEV canonical markers), with RATregs being the best producers of sEV. In terms of *in vitro* suppression activity, all sEV inhibit CD4+ and CD8+ T cells proliferation, whereas no conclusive results were obtained in terms of cytokine secretion as the variability between experiments was high. Suppression assays performed in the presence of nTregs-, iTregs- and RATregs-derived sEV indicate that CD4+ T cells go into apoptosis in the presence of sEV obtained from nTregs and RATregs. Furthermore, proteomic analysis of nTregs-, iTregs- and RATregs-derived sEV show the presence of common immune regulatory proteins and reveals the presence of apoptosis-related proteins such as Granzymes and Perforins.

This study demonstrates that sEV from nTregs, iTregs and RATregs produce sEV with similar size and markers, T cell suppressive activity, and a cytotoxic effect. Interestingly, only sEV isolated from nTregs and RATregs -and not iTregs- enhance cell death. Furthermore, it indicates that sEV protein content differs depending on the cellular source of obtention. This protein cargo may translate into -different- functional effects on the target cell, including immune regulation via cell death/cytotoxicity.

## Materials and methods

### Mice

Female and male mice of 6–8 weeks old were used. FoxP3^GFP^ were from The Jackson Laboratory (Maine, USA). All mice were under pathogen-free conditions at a room temperature of 22 °C with 12h light/dark cycle and food and water ad libitum. The animal facility is located at the Facultad de Medicina, Universidad de los Andes, Santiago, Chile. All procedures were performed in accordance with the bioethics committee guidelines from Universidad de los Andes and the National Research and Development Agency of Chile (ANID) and carried out after the approval of the experimental protocol (#CEC2021017).

### Cell culture

CD4+CD25^high^ T cells (nTregs) and CD4+CD25- T cells were isolated from spleen and lymph nodes (LN) of FoxP3^GFP^ mice. In brief, nTregs were obtained using the Murine CD4+CD25+ T regulatory Cell Isolation Kit (Cat n°130-091-041, Miltenyi Biotec, CA, USA) following the provider´s instructions. After the second step, the “negative” fraction (CD4+CD25- T cells) was conserved to induce iTregs and RATregs, whereas nTregs were obtained after flushing the column during the last step. 1 x 10^6^/mL of nTregs were cultured in the presence of polyclonal activation given by plate-bound 5 μg/mL antiCD3 (clone 145-2C11, BioXcell, USA) and 2 μg/mL antiCD28 (clone 37.51, BioLegend, NJ, USA), for 72h in a 24-well plate and incubated at 37 °C, 5% CO_2_. For iTregs and RATregs induction, a mix of 10 μg/mL plate-bound antiCD3 (clone 145-2C11, BioXcell, USA) and 1 μg/mL antiCD28 (clone 37.51, BioLegend, NJ, USA) in PBS 1X was added to 24-well plates and incubated at 37 °C, 5% CO_2_. After mix removal, 2 x 10^5^ cells/well were seeded in 1 mL of cRPMI [which contains penicillin/streptomycin 1% (Corning, NY, USA), HEPES 1% (Gibco, MD, USA), β-mercaptoethanol 0,1% (Sigma, MO, USA), 10% of sEV-free FBS (Gibco, MD, USA), 100 UI/mL of human IL-2 (#200-02, Peprotech, NJ, USA) and 10 ng/mL of murine TGF-β1 (#100-21, Peprotech, NJ, USA)] for iTregs induction and the same cytokines plus 10nM all-*trans* retinoic acid (ATRA, #R2625, Sigma-Aldrich, Massachusetts, USA) for RATregs. After three (nTregs) and five (iTregs/RATregs) days in culture, cells were harvested for phenotype analysis by flow cytometry, and supernatants were collected for sEV isolation.

### Flow cytometry

Cell samples were stained with antibodies against: CD4 (clone RM4-5), CD25 (clone PC61) and Nrp1 (clone 3E12) (all from BioLegend, CA, USA), and CD73 (clone TY/11.8. from eBioscience, San Diego, CA, USA). All antibodies were conjugated with PE, PerCP-Cy7, APC or APC-Cy7. Data acquisition was performed using a BD FACSCanto II cytometer (BD Biosciences, CA, USA), and data were analyzed using FlowJo software (Tree Star, OH, USA).

### sEV preparation and isolation

sEVs were isolated using differential centrifugation. In brief, a first step using 300 g for 5 min, followed by 2–000 g for 20 min were applied for cell removal and elimination of dead cells, respectively. Then, supernatants (SN) were centrifuged twice at 10–000 g for 30 min, followed by a last step applying 100–000 g for 90 min. The pellets were resuspended in ~100 μL of exo-free cRPMI, which were subjected to sEV enrichment using IZON Columns (qEVoriginal/35 nm Gen 2, Christchurch, New Zealand) following the provider´s instructions.

### Nanoparticle tracking analysis

​​NTA was performed using a NanoSight NS300 (Malvern Instruments, UK) equipped with a 532 nm laser and a 565 nm LP filter. Three videos of 30 sec were recorded of each sample, with temperature monitored along the readings (25 °C). Videos recorded for each sample were analyzed with the Nanosight NS300 NTA software to determine size and concentration of particles samples. The Nanosight instrument was calibrated using 100 nm diameter polystyrene latex beads and 100 nm diameter 532-nm green fluorescence standards (all from Malvern Panalytical, UK).

### Suppression assay

Splenocytes were obtained from murine spleens and labeled with 5 μM CellTrace™ Violet (CTV, ThermoFisher Scientific, MA, USA). 2 x 10^5^ responder cells were polyclonally activated with soluble 1 μg/mL αCD3 alone (control) or including 1 x 10^6^, 1 x 10^7^ or 1 x 10^8^ sEV particles derived from nTregs, iTregs or RATregs, in round-bottom 96-well plates containing 200 μL of sEV-free cRPMI media for 72 h. T cell proliferation was analyzed tracking CTV dilution by flow cytometry. Suppression was calculated as previously described (12). In short, we used the following formula: % Suppression = (1 – DI_sEVTreg_/DI_Tresp_) x 100% (where DI_sEVTreg_ stands for the division index of responder cells with sEV Treg, and DI_Tresp_ stands for the division index of responder cells activated without treatment).

### Apoptosis assay

Total splenocytes were cultured in round-bottom 96-well plates, containing 200 μL of sEV-free cRPMI media with or without sEV (1x10^8^ particles) from nTregs, iTregs and RATregs. After 72h in culture, apoptosis was determined by tracking annexin and PI staining by flow cytometry using the BD Pharmingen™ FITC Annexin V Apoptosis Detection Kit I (Catalog No: 556547, San José, CA, USA).

### ELISA

Supernatants from suppression assays were collected and stored at -80 °C for cytokine quantification by ELISA (sandwich) test. Pure and Biotin-conjugated antibodies for the following cytokines were purchased from Biolegend (San Diego, CA, USA): IFN-γ (clone R4-6A2), IL-17 (clone TC11-8H4), and IL-10 (clone JES5-16E3) in addition to the corresponding recombinant murine cytokines for the standard curves (all from Peprotech, NJ, USA).

### Mass spectrometric analysis of sEV content

sEV proteins were separated using polyacrylamide gradient gel electrophoresis. Each lane was divided into 3 sections to perform in-gel digestion according to Kolodziej et al. (13). LC-MS/MS was performed on a hybrid dual pressure linear ion trap/orbitrap mass spectrometer (LTQ Orbitrap Velos Pro, Thermo Scientific, San Jose, CA, USA) equipped with an Ultimate 3000-nLC Ultra HPLC (Thermo Scientific, San Jose, CA, USA). Dried peptide fractions were dissolved in 10 μL 0.1% TFA and subjected to a 200cm µPAC™ RP C18-csA column (PharmaFluidics, Ghent, Belgium). Separation was achieved by applying a gradient from 2% ACN to 35% ACN in 0.1% formic acid (FA) over a 180 min gradient at a flow rate of 1.6 µL/min. The LTQ Orbitrap Velos Pro MS exclusively used CID-fragmentation when acquiring MS/MS spectra, consisting of an orbitrap full MS scan followed by up to 20 LTQ MS/MS experiments (TOP20) on the most abundant ions detected in the full MS scan. The essential MS settings were as follows: full MS (FTMS; resolution 60,000; m/z range 400–2000); MS/MS (Linear Trap; minimum signal threshold 500; isolation width 2 Da; dynamic exclusion time setting 30 s; singly charged ions were excluded from selection). Normalized collision energy was set to 35%, and the activation time was set to 10 ms.

Raw data processing and protein identification of the high resolution orbitrap datasets were performed with *de novo* sequencing algorithms of PEAKS Studio 8.0 (Bioinformatics Solutions Inc., Waterloo, Canada) using the SwissProt database. The false discovery rate was set to <1%. Label free quantification was achieved by using PROGENESIS QI for proteomics (Nonlinear dynamics/Waters).

### Bioinformatics analysis

The differentially expressed proteins of each sEV sample were analyzed by Venn diagrams using the website https://bioinformatics.psb.ugent.be/webtools/Venn/. Gene ontology analysis, Functional Annotation, Gene Functional Classification and Gene ID Conversion were performed using GeneOntology Consortium (14–16) and PANTHER version 18.0 (16, 17). Heatmaps of protein expression data were performed using Prism 8 (GraphPad), with the data extracted from de mass spectrometry analysis.

### Statistical analysis

Statistical analysis was performed using Prism software. Mann-Whitney test was used. P values < 0.05 were considered statistically significant, and the significance were indicated in the figure legend according to each figure.

## Results

### Production of sEV from nTregs, iTregs and RATregs, and their suppressive function

**Figure 1:**
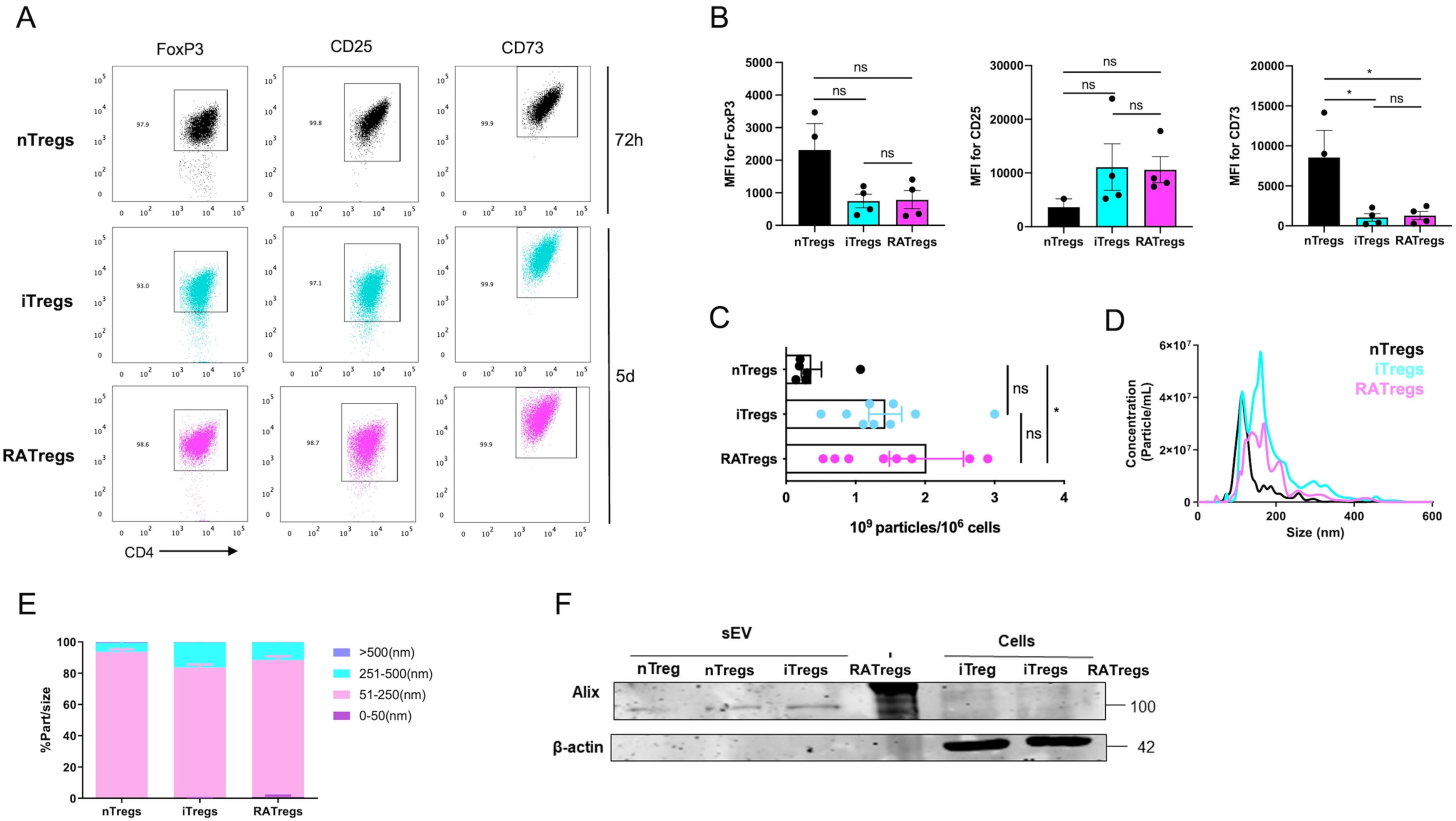
nTregs, iTregs and RATregs produce sEV. nTregs were obtained from spleens of FoxP3^GFP^ mice using the CD4+CD25+ Miltenyi kit. 10^6^/mL of nTregs were cultured in the presence of polyclonal activation (αCD3/αCD28) for 72h. To produce iTregs, the CD4+CD25- T cells fraction obtained from the nTregs purification was used. iTregs were obtained after 5 days in culture in the presence of polyclonal activation, IL-2 and TGF-β, whereas RATregs had retinoic acid (RA) added in addition to IL-2 and TGF-β. Tregs characterization included FoxP3, CD25 and CD73 expression analysis by flow cytometry **(A)** Representative dot plots displaying the phenotype of nTregs (black), iTregs (blue) and RATregs (pink). **(B)** Bar plots depicting the MFI for FoxP3, CD25 and CD73 from all Tregs subsets. **(C)** Graph showing the number of sEV per 10^6^ cells obtained from nTregs, iTregs and RATregs. **(D)** Size distribution of sEV obtained from nTregs, iTregs and RATregs determined by NTA. **(E)** Abundance of sEV by size range also determined by NTA. **(F)** Western blot showing Alix as marker for sEV and β-actin as a marker for (Tregs) cells. A-E. Each experiment was performed 4–8 times (each dot corresponds to a single cell culture). **(F)** Western blot was performed 2 times. *p < 0.05 according to One-way ANOVA test; ns, not significant. Bar lines represent Mean ± SEM.

With the purpose of characterizing and comparing sEV obtained from different Tregs subsets, we isolated nTregs and produced induced Tregs in the presence of TGF-β without (iTregs) and with ATRA (RATregs) as described in the method section. Freshly isolated nTregs were >92% FoxP3^GFP+^, >90% CD25+ and >95% CD73+; conversely, conventional CD4+ T cells showed ˜3% of FoxP3, <1% expressed CD25 and ˜45% expressed CD73, **Supplementary Figure 1**. After *in vitro* culture of nTregs for 72h and the differentiation of iTregs and RATregs for 5 days, we found that >90% of cells remained FoxP3+, >88% CD25+ and >89% CD73+, **Figure 1A**. In terms of mean fluorescence intensity (MFI), nTregs showed the highest expression of FoxP3 (MFI ˜2 000) and CD73 (MFI ˜8 000), whereas CD25 expression was reduced (MFI ˜4 000) in comparison with iTregs and RATregs (MFI ˜10–000 for both iTregs and RATregs), although no statistical difference was obtained, **Figure 1B**. After confirming Tregs canonical phenotype, we continued with sEV isolation and characterization. To secure a good enrichment of sEV, we characterized each eluted fraction (from the isolation column) measuring particle size by NTA and presence of Alix and α-tubulin by western blot, **Supplementary Figure 2**. This previous test allowed us to pool fractions 2 to 8 resulting in good number and concentration of particles. As shown in **Figure 1C**, NTA indicated that RATregs produce sEV particles per million cells (~2x10^9^) than nTregs (~0,5x10^9^). With respect to sEV size, nTregs release sEV with an average size of 110 nm whereas iTregs and RATregs produce sEV of 160 nm, **Figure 1D**. The overall size distribution for the three types of sEV shows that ˜85-95% of the particles fall within the 51–250 nm range, **Figure 1E**. A closer examination of their sizes reveals that ˜20% of iTregs-derived sEV, and ˜10% of RATregs-derived sEV, fall within the 251–500 nm range. In addition, western blot analysis showed that nTregs-, iTregs- and RATregs-derived sEV contain Alix (marker for endosomal origin) but no β-actin (18, 19), **Figure 1F**. Taking all together, we can conclude that nTregs, iTregs and RATregs produce sEV containing Alix, and that nTregs and RATregs produce vesicles with similar size in comparison with iTregs, which display a heterogeneous pool of particles.

**Figure 2:**
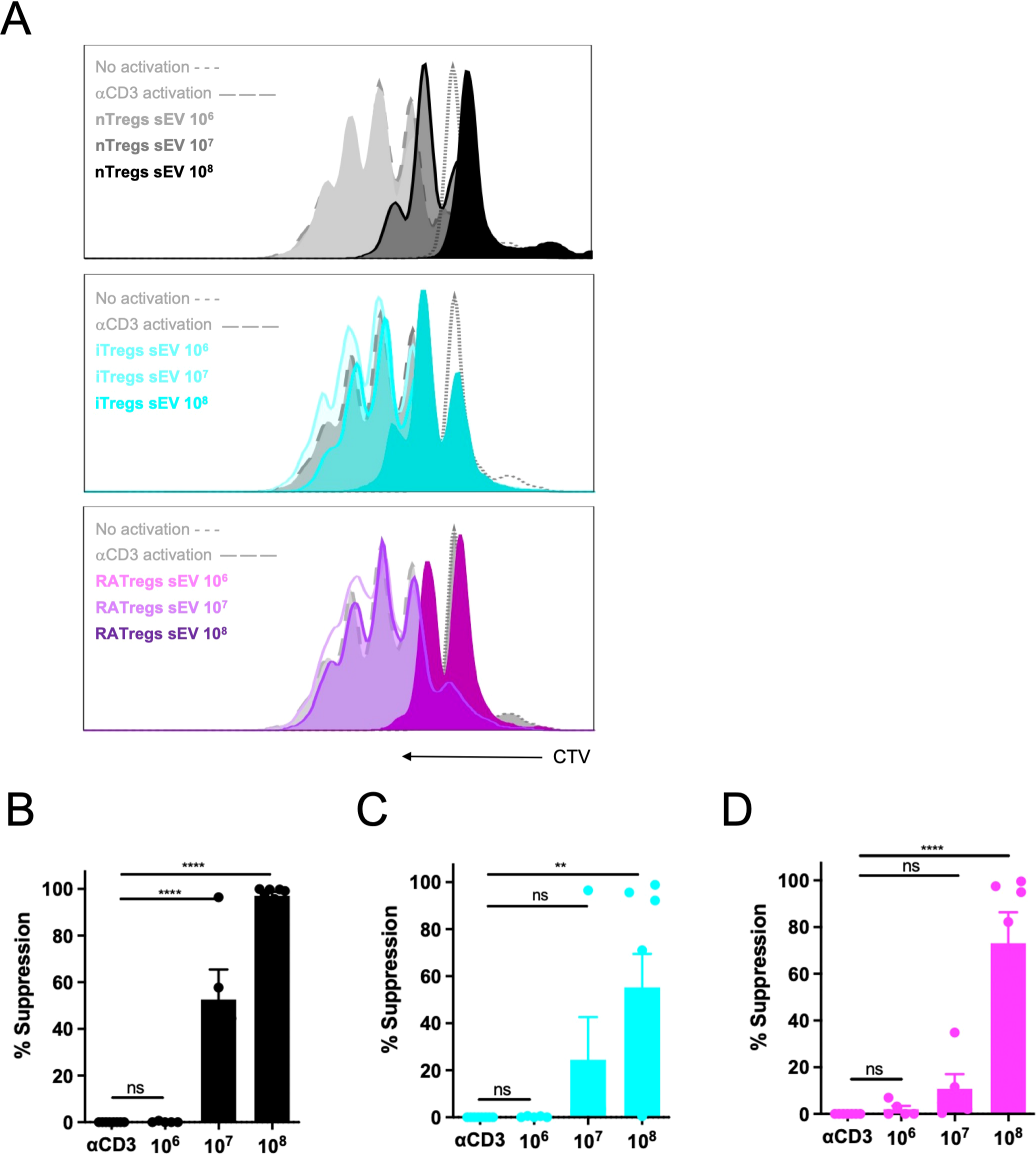
sEV obtained from nTregs, iTregs and RATregs suppress T cell proliferation Suppression assay was performed as detailed in material and methods section, and analysis was performed by flow cytometry. **(A)** Representative histograms depicting CTV dilution (proliferation) of CD4+ T cells polyclonally activated (αCD3) without or with different amounts of sEV from Tregs subsets (10^6^, 10^7^ or 10^8^ sEV). Bar graphs displaying the percentage of suppression when sEV were obtained from nTregs **(B)**, iTregs **(C)** and RATregs **(D)**. Each experiment was performed 5 times (each dot corresponds to a single cell culture). **p < 0.01, ****p < 0.0001 according to One-way ANOVA test; ns, not significant. Bar lines represent Mean ± SEM.

Next, we tested Tregs-derived sEV function using a suppression assay *in vitro*. Total splenocytes were labeled with CTV and activated polyclonally with αCD3 for 72h in the presence of the three types of sEV at different quantities. As shown in **Figures 2A–D**, all types of sEV display T cell inhibitory activity: sEV obtained from nTregs reach ˜100% of suppression at the high (10^8^ particles) dose, and ˜50% of suppression with the medium (107) dose, **Figures 2A, B**; sEV obtained from iTregs reach ˜60% of suppression with the high dose and ˜20% of suppression with the medium dose, **Figure 2A** and C; and sEV obtained from RATregs reach ˜70% of suppression with the high dose and only ˜10% of suppression with the medium dose, **Figure 2A, D**. The low dose of 10^6^ sEV was ineffective in suppressing T cell proliferation, **Figures 2A–D**. The same trend was found for CD8+ T cells, in which sEV of nTregs origin resulted better suppressors than those isolated from iTregs/RATregs (**Supplementary Figure 3**). To complement the modulatory effect of sEV, we performed cytokine quantification for IFN-γ, IL-17 and IL-10, but no conclusive results were obtained. In conclusion, sEV obtained from nTregs, iTregs and RATregs suppress CD4+ and CD8+ T cells proliferation *in vitro*, with sEV from nTregs displaying the highest effect at the highest dose. Because the effect of sEV on cytokine production seems different between the three types of sEV tested, we could postulate that sEV content may differ depending on the cellular origin. In other words, sEV obtained from nTregs may be composed by different molecules than sEV obtained from induced Tregs (with or without RA).

### sEV obtained from nTregs and RATregs enhance cell death on target cells

**Figure 3:**
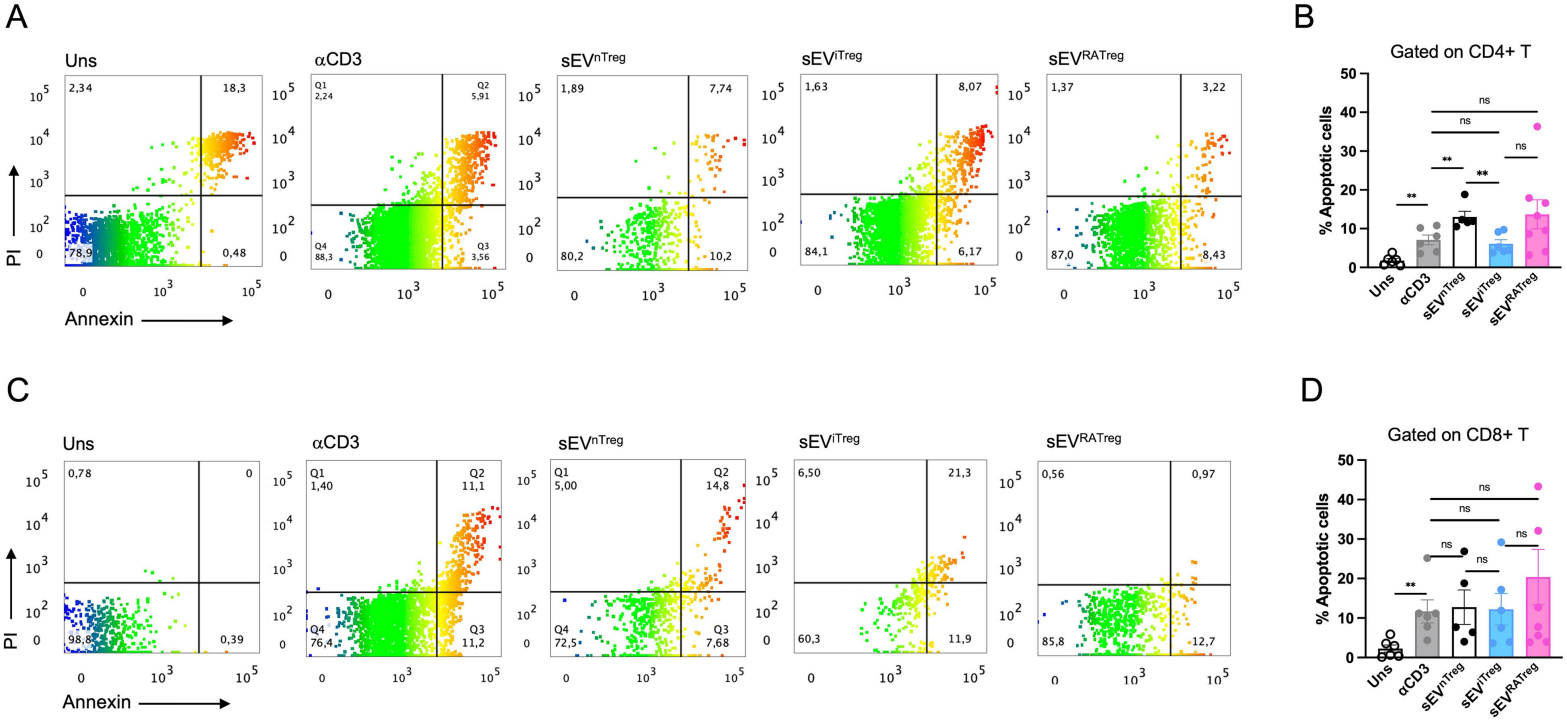
Tregs-derived sEV favor cytotoxic activity Tregs subsets and sEV were obtained as described in materials and methods section. CTV-labeled splenocytes were activated with αCD3 in the presence of 10^8^ sEV. Apoptosis was determined by flow cytometry identifying Annexin+PI- cells on CD4+ T cells population. As indicated, an αGrzB antibody was included in some conditions. **(A)** Representative dot plot showing the frequencies of live (Annexin-PI-), apoptotic (Annexin+PI-) and dead (Annexin+PI+) CD4+ T cells. **(B)** Bar graph depicting the frequencies of live CD4+ T cells unstimulated (Uns), activated (αCD3) alone or in the presence of the three types of sEV. **(C)** Bar graph depicting the frequencies of apoptotic or dead **(D)** CD4+ T cells unstimulated (Uns), activated (αCD3) alone or in the presence of the three types of sEV. The experiment was performed 5 times (for conditions with sEV, each dot corresponds to a different sEV preparation). *p < 0.05, **p < 0.01, ***p < 0.001 according to One-way ANOVA test; ns, not significant. Bar lines represent Mean ± SEM.

During the analysis of the suppression assays, we realized that cell death was taking place on some the conditions treated with sEV. Thus, we evaluated whether sEV could induce cell death on target cells by using the Annexin-V/PI assay. Total splenocytes were cultured for 72h in absence of activation (unstimulated), and in the presence of polyclonal activation with or without 10^8^ particles of sEV obtained from nTregs, iTregs and RATregs. We observed that activation increased the frequencies of Annexin^high^PI- (early-stage apoptosis) CD4+ T cells (from ˜1% in unstimulated cells to ˜5% in the αCD3 condition), but the addition of sEV from nTregs and RATregs increased these frequencies to ˜12%. Interestingly, sEV from iTregs did not have this effect and behave as the αCD3 condition, **Figures 3A, B**. When analyzing CD8+ T cells, sEV obtained from nTregs and iTregs triggered the same levels of early-stage apoptosis as the activation control alone (˜10%). Although not statistically significant, sEV from RATregs seem to enhance early-stage apoptosis on CD8+ T cells, **Figures 3C, D**. The possibility that the cellular source may be contributing with apoptotic bodies (due to cells undergoing apoptosis during cell differentiation/culture) was ruled out since iTregs and RATregs showed ≥93% of viability, ˜2% of early-stage apoptotic cells and ˜5% of late-stage apoptosis/necrotic cells at the end of the differentiation culture, **Supplementary Figure 5**. Overall, these data indicate that Tregs-derived sEV favor cytotoxicity -direct or indirectly- on target cells, discriminating sEV of nTregs and RATregs origin from iTregs.

### Protein characterization reveals enrichment of apoptosis-related molecules on sEV from Tregs subsets

**Figure 4:**
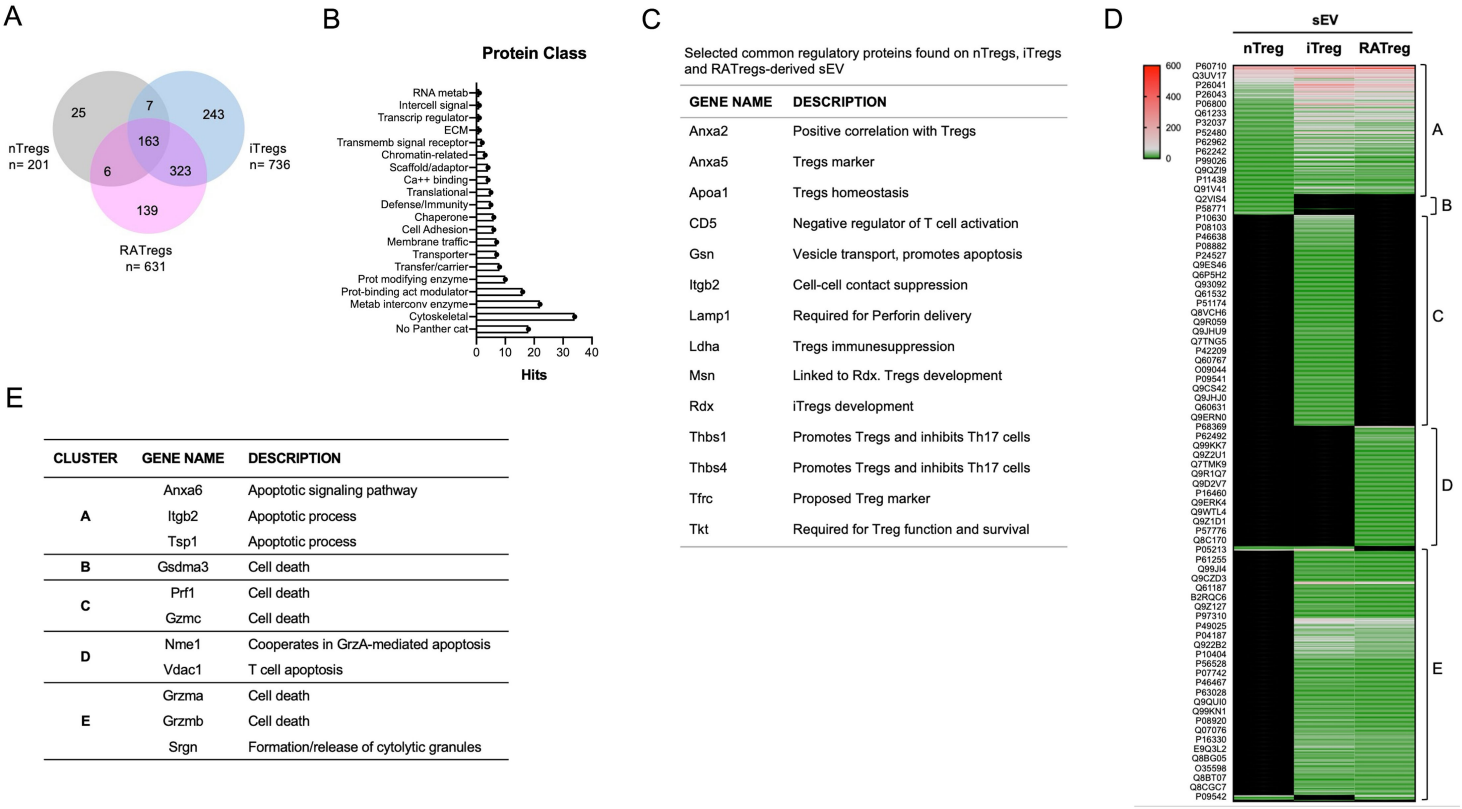
Proteomic analysis on sEV obtained from different Tregs subsets reveals common immune regulatory molecules sEV prepared from nTregs, iTregs and RATregs were subjected to proteomic analysis as described in materials and method section (n=3 independent sEV batches per Tregs type). **(A)** Venn diagram showing proteins present on each type of sEV or proteins shared between two or three types. **(B)** Plot displaying the Protein Class category for the proteins found in common between the three types of sEV using the PANTHER website. **(C)** Table showing a selection of proteins involved on Tregs phenotype or function. **(D)** Heatmap showing the abundance of proteins for each type of sEV (or their combination) and the resulting clusters. Color range indicates high (red) to low (green) abundance. **(E)** Table showing a selection of cell death-related proteins found on clusters A to E.

Previous data from our laboratory demonstrated that sEV isolated from nTregs are enriched on regulatory molecules such as Nrp1, CD73 and CD25 (20). Hence, to investigate whether sEV protein content could account for the apoptotic effect on T cells, we performed label‐free liquid chromatography‐tandem mass spectrometry (LC‐MS/MS) ‐based proteomics to determine their composition and potential distinctive profiles. The proteomes of three biological replicates of sEV obtained from nTregs, iTregs and RATregs were compared obtaining 201, 736 and 631 proteins, respectively. The Venn diagram shows that 163 proteins are common among the three types of sEV, whereas 25, 243 and 139 proteins were exclusively detected on nTregs-sEV, iTregs-sEV and RATregs-sEV, respectively, **Figure 4A**. Lists with all proteins can be accessed in **Supplementary Tables I-V**. Gene Ontology (GO) enrichment analysis was performed considering the 163 “common” proteins using Panther GenOntology Consortium. Even though 18 proteins (11%) were not categorized, the majority were clustered in the “Cytoskeletal” class (20.9%), “Metabolism interconversion enzymes” class (13.5%) and “Protein-binding activation modulator” class (9.8%), finding some proteins in the “Defense/Immunity” class as well (3.1%), **Figure 4B**. Alternatively, we carried out a detailed analysis searching for the function of these “common” proteins on the literature, finding several molecules involved in Tregs development, function and survival, such as Apolipoprotein AI (Apoa1) (21), Integrin b2 (Itgb2) (22), Lactate Dehydrogenase-A (Ldha) (23), Thrombospondin 1 (Thbs1) (24), Transferrin Receptor (Tfrc) (25), among others, **Figure 4C**. As an additional way to visualize the data, we built a heatmap using GraphPad, which displays 5 major clusters (A to E): one for the common molecules (cluster A), other clusters with specific molecules per Tregs subset (clusters B-D), and a cluster showing molecules shared between iTregs and RATregs-derived sEV (cluster E), **Figure 4D**. When studying cluster A, we recognized molecules involved in cell death, such as Annexin 6 (Anxa6) (26), Integrin β2 (Itgb2) (27) and Thrombospondin-1 (Tsp1) (28). Furthermore, Gasdermin-3 (Gdsma3) (29) was enriched in cluster B; Perforin-1 (Prf 1) and Granzyme C (GrzC) (30) were enriched in cluster C; Nucleoside diphosphate kinase A (Nme1) (31) and Voltage-dependent anion channel (Vdac) (32) were enriched in cluster D; and Granzyme A (GrzA), Granzyme B (GrzB) (30) and Serglycin (Srgn) (33) were found in cluster E, **Figure 4E**. These results indicate that the presence of these proteins on sEV from Tregs may play a role in the previously observed sEV-mediated cytotoxicity (**Figure 3**).

Thus, we demonstrate that Tregs subsets produce sEV with different protein content, underscoring the presence of cell death-related proteins that could mediate this process as a suppressive mechanism.

## Discussion

Tregs release sEV as one of their suppressive mechanisms (34–36), but a comparison between their origin (natural versus induced) and function has not been reported. Here, we isolated sEV from nTregs, and from iTregs produced *in vitro* in the presence of TGF-β alone or with RA (RATregs). Our data indicates that all three types of Tregs secrete sEV with similar size and contain the canonical sEV marker Alix, **Figure 1**. In terms of particles number, RATregs produce more sEV than the other two types, suggesting that the inclusion of RA may impact on CD4+ T cell activation status since sEV production by T cells depends on this cellular state (37, 38). Furthermore, *in vitro* experiments showed that sEV isolated from nTregs, iTregs and RATregs inhibit CD4+ T and CD8+ T cell proliferation, **Figure 2**. This observation agrees with previous studies in which sEV isolated from nTregs (35, 36, 39), iTregs (9), and RATregs (10) display T cell inhibitory effects. This inhibition may include the transfer of modulatory miRNAs to target cells (9, 35), the activity of CD73 (10, 36), or the presence of Nrp1 (39) on sEV. Also, T cell inhibitory function by all three types of sEV resulted dose-dependent, where the best T cell inhibition was observed with 10^8^ particles of sEV, **Figure 2**. To complement on the mechanism of action of these sEV, we measured IFN-γ, IL-17 and IL-10 in suppression assays supernatants, but no definitive conclusions could be made from these data due to the variability of the results **Supplementary Figure 4**. Importantly, in addition to sEV inhibitory effect on T cell proliferation, this study reveals that not only nTregs induce cytotoxicity via sEV, as previously reported (40), but also iTregs and RATregs, **Figure 3**. In fact, cytotoxicity occurs with the inclusion of sEV from nTregs, iTregs or RATregs, but it is enhanced in the presence of sEV from nTregs and RATregs, **Figure 3**. With the purpose to find a potential association between sEV-induced cell death and molecular content, we proceeded with proteomic analysis to decipher sEV composition. As shown in **Figures 4A–C**, sEV from nTregs, iTregs and RATregs contain proteins in common, some of which correspond to molecules involved on Tregs biology: Anxa2, a protein linked to bad prognosis in cancer (41), Anxa5, a molecule that identifies a subset of Tregs (42), Apoa1, a protein that promotes Tregs differentiation (21), Ldha, an enzyme that may play a role on Tregs differentiation/function in the tumor context (23, 43), Thbs1, a protein involved on Tregs differentiation (24), and Tfrc, a receptor controlling Tregs expansion and metabolism (25), among others. Further analysis revealed 5 clusters that contain enriched proteins involved in cell death as Anxa6, Itgb2 and Tsp1 (cluster A), Gdsma3 (cluster B), Prf1 and Grzmc (cluster C), Nme1 and Vdac1 (cluster D); and Grzma, Grzmb and Srgn (cluster E), **Figures 4D, E**. Anxa6 is a protein involved on several cell death pathways and on T cells activation and proliferation (26, 44), Itgb2 is an integrin β-chain protein that interacts with α-chains to form heterodimers (27), Tsp1 is a secreted matricellular glycoprotein with multiple functions on immune cells (28). Gdsma3 is a member of the Gasdermin family, a group of pore-forming proteins related to pyroptosis (a pro-inflammatory process of cell death) with scarce number of reports involving T cells biology (29, 45), Nme1 is a tumor suppressor gene with influence on apoptosis and T cell biology, and Vdac1 is a molecule that forms pores (a porin) in the outer membrane of the mitochondria, and it has been implicated in processes such as apoptosis and cytokine release, among others (46). On the contrary, granzymes such as A, B and C, and even Prf1 have been reported as mediators of Tregs function (6). On this regard, cytotoxic function for Tregs was first mentioned in 2004, where human Tr1 cells (non-FoxP3 Tregs) up-regulate GrzB and Prf1 expression upon TCR activation, and mediate target cell killing in a FasL-independent manner (47). Similar results have been found on human CD4+CD25^hi^FoxP3+ nTregs, which predominantly express GrzA and Prf1 upon activation, and drive cell killing (48).

Thus, this report demonstrates that different Tregs subsets produce sEV that favor cytotoxic activity, whose mechanism may differ depending on the source of Tregs. How exactly cell death is triggered by sEV is a pending question: one possibility is that target cells (effector T cells) uptake Tregs-derived sEV (35) resulting in the incorporation of cell death-related molecules, among other mediators, and initiation of the apoptotic process. Alternatively, Tregs-derived sEV could affect other cells that become activated and consequently induce cell death on T cells. It is important to note that the possibility of Tregs dying in culture and releasing apoptotic bodies was discarded as cell viability was >93% at the end of cultures, **Supplementary Figure 5**. Although this report is not exempted of limitations, such as high variability of *in vitro* (ELISA) data, the failure to identify the pathway involved in sEV-induced cell death, and the lack of an *in vivo* model to evaluate the physiological significance of our observations, we still believe that the results contribute to the understanding of Tregs mechanisms of suppression and the involvement of sEV release.

Given that all three types of sEV showed suppressor function, and considering the challenges posed by the small number of sEV prepared from nTregs - along with their difficult isolation, expansion, and maintenance *in vitro* - iTregs and RATregs emerge as promising cellular sources of sEV for therapeutical interventions, with the pending task of deciphering the implications of the cytotoxicity levels observed by sEV from these cells.

## Data Availability

The original contributions presented in the study are included in the article/**Supplementary Material**. Further inquiries can be directed to the corresponding author.

## Supplementary material

Supplementary Figure 1 Phenotype of nTregs and conventional CD4+ T cells at day 0 of culture Representative plot showing the expression of FoxP3, CD25 and CD73 on nTregs and conventional CD4+ T cells (CD25-FoxP3-) at day 0 of culture.

Supplementary Figure 2 Determination of sEV content on elusion fractions obtained from the size-exclusion columns A. Size determination by NTA of particles obtained in each fraction during sEV enrichment. C. Western blot for determining the presence of Alix and α-Tubulin in each fraction during sEV enrichment.

Supplementary Figure 3 Suppression of CD8+ T cell proliferation by sEV obtained from nTregs, iTregs and RATregs Suppression assay was performed as detailed in materials and methods section, and analysis was performed by flow cytometry. A. Bar graph depicting the percentage of suppression on CD8+ T cells when sEV were obtained from nTregs (A), iTregs (B) and RATregs (C) using 10^6^, 10^7^ or 10^8^ sEV as indicated. ** p < 0.01, *** p < 0.001, **** p < 0.0001 according to according to One-way ANOVA test; ns, not significant. Bar lines represent Mean ± SEM.

Supplementary Figure 4 sEV obtained from Tregs subsets differentially modulate cytokine release. Supernatants collected from the suppression assays were subjected to ELISA. A. Violin plots showing the concentrations of IFN-γ (A), IL-17A (B) ad IL-10 (C) produced by splenocytes cultured in the presence of sEV obtained from nTregs (black), iTregs (blue) and RATregs (pink). Violin plots lines represent the Median.

Supplementary Figure 5 Viability of iTregs and RATregs at day 5 of culture Tregs subsets were obtained as described in materials and methods section. Cell viability, apoptosis and necrosis were determined by flow cytometry. A. Representative dot plot showing the frequencies of live (Annexin -PI-), apoptotic (Annexin+PI-) and dead (Annexin+PI+) iTregs (left) and RATregs (right). B. Bar graph depicting the frequencies of live, apoptotic (C) and dead (D) iTregs and RATregs. The experiment was performed 3 times (each dot corresponds to a single cell culture). ns, not significant. Bar lines represent Mean ± SEM.

Supplementary Figure 6 Gating strategies for suppression and apoptosis assays A. Representative dot plot showing the gating strategy used for determining the frequencies of live, early-stage apoptosis and late-stage apoptosis/necrosis. B. Representative dot plots showing the gating strategy used for determining CD4+ T cells proliferation (CTV dilution).
