# Supplementary material for: CD4+FoxP3+ T regulatory cells subsets release small extracellular vesicles containing cell death-related proteins as potential mechanism of T cell suppression
Source: Front Immunol. 2026 Apr 21;17:1777669. doi: 10.3389/fimmu.2026.1777669 (PMC13139319; doi:10.3389/fimmu.2026.1777669)
Supplement: Supplementary Figure 1 — Phenotype of nTregs and conventional CD4+ T cells at day 0 of culture Representative plot showing the expression of FoxP3, CD25 and CD73 on nTregs and conventional CD4+ T cells (CD25-FoxP3-) at day 0 of culture. [file Presentation1.pptx]

## Slide 1
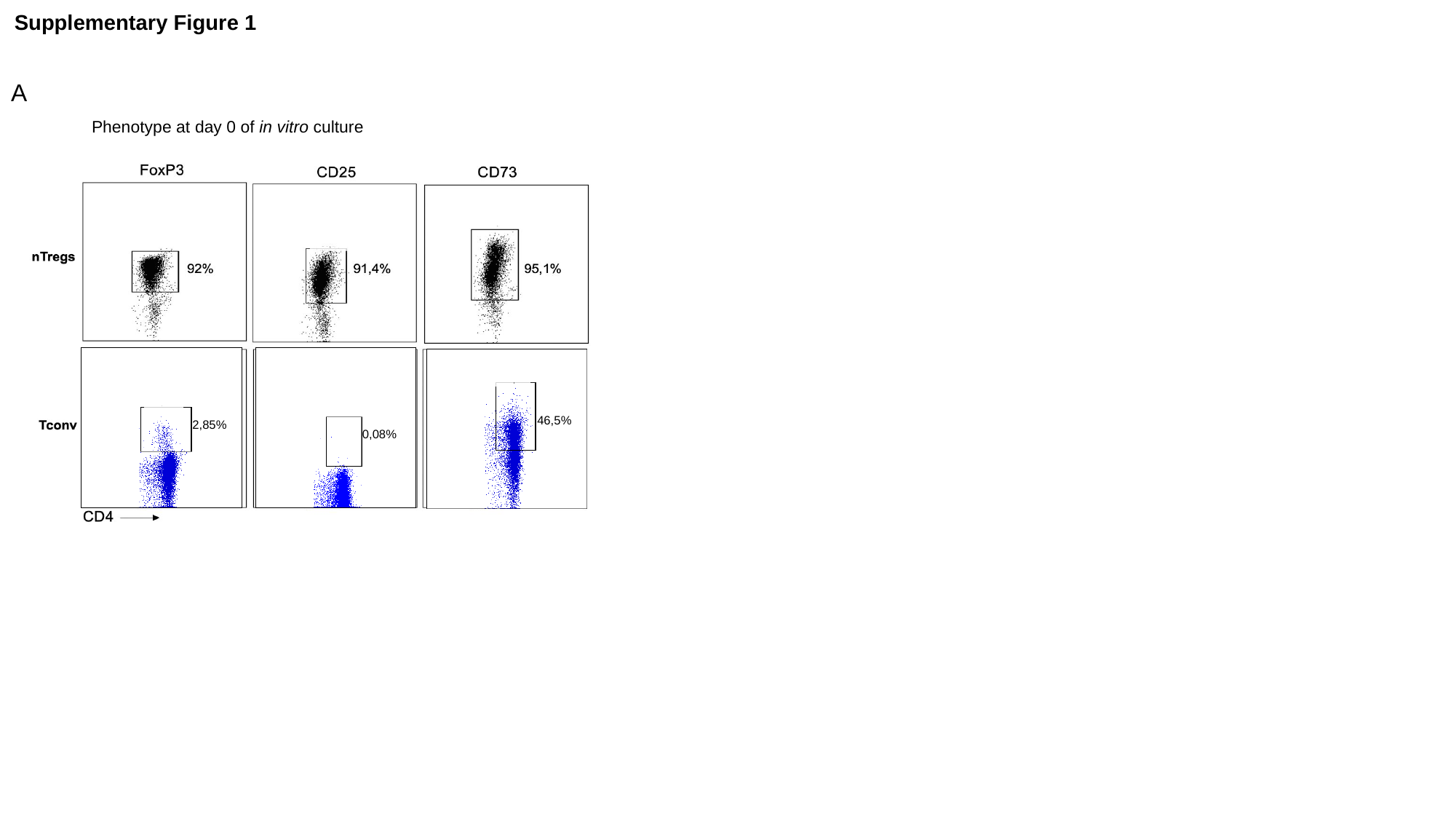

Supplementary Figure 1
A
Phenotype at day 0 of in vitro culture
46,5%
2,85%
0,08%

## Slide 2
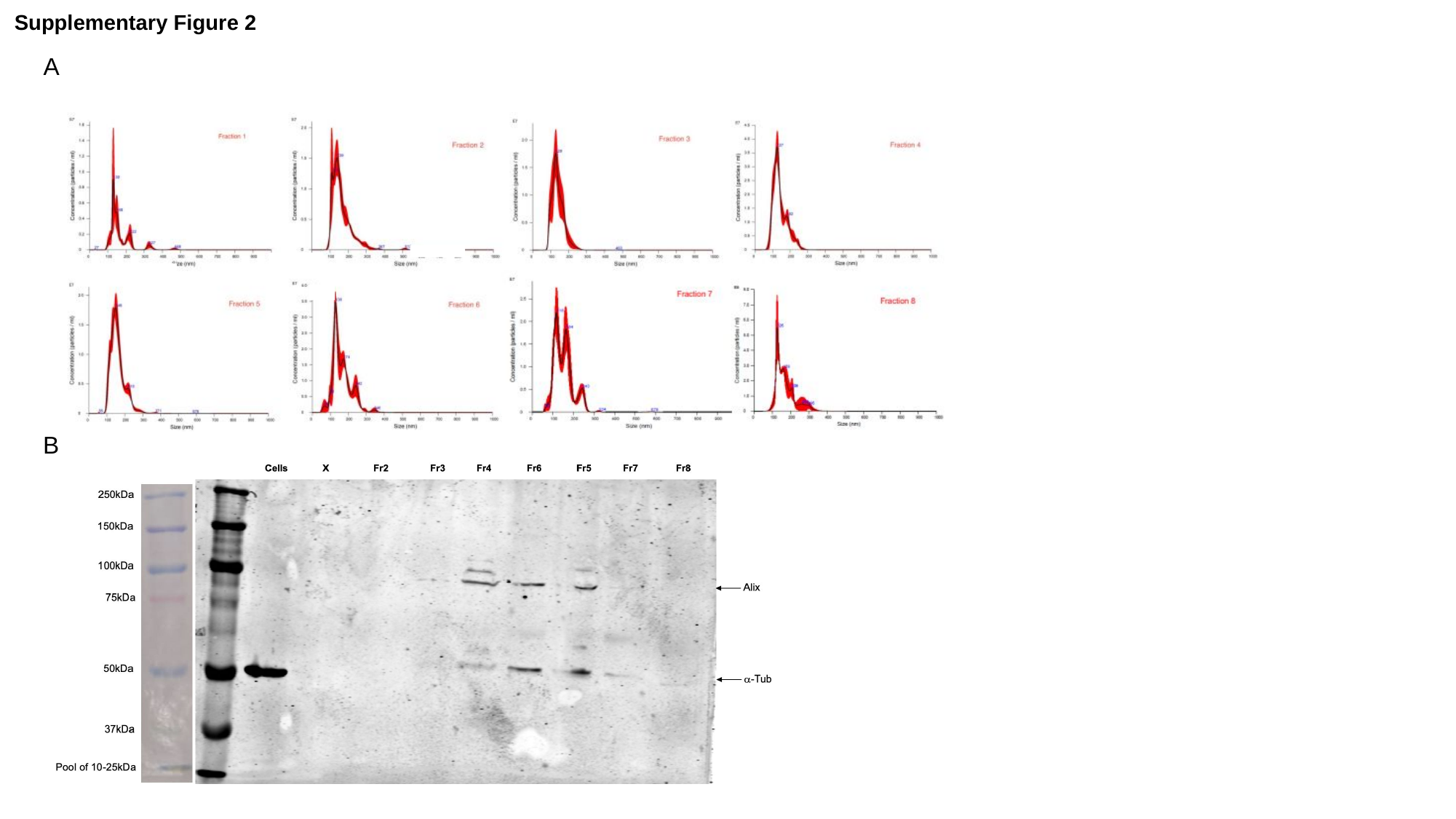

Supplementary Figure 2
A
B

## Slide 3
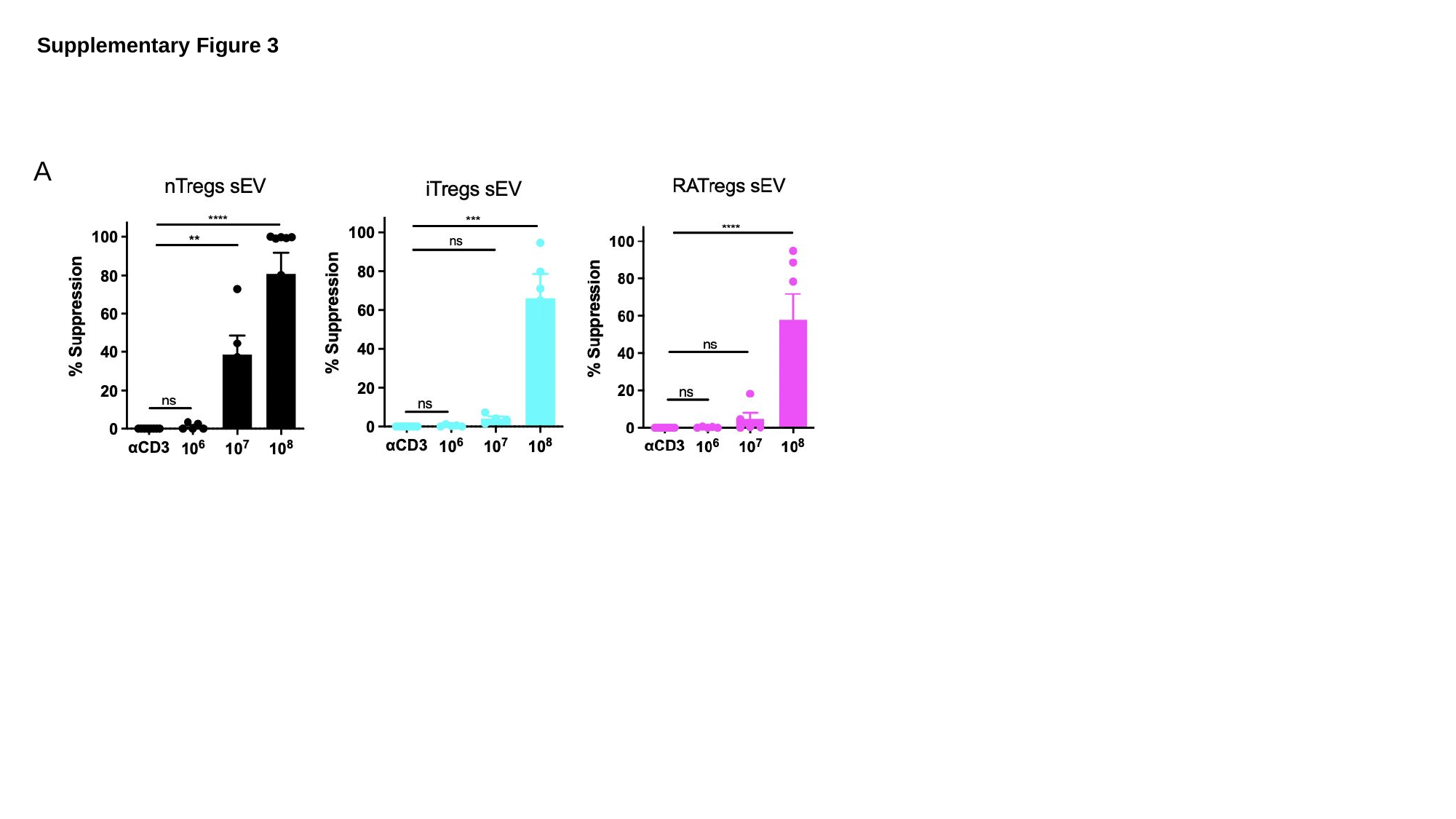

Supplementary Figure 3
A

## Slide 4
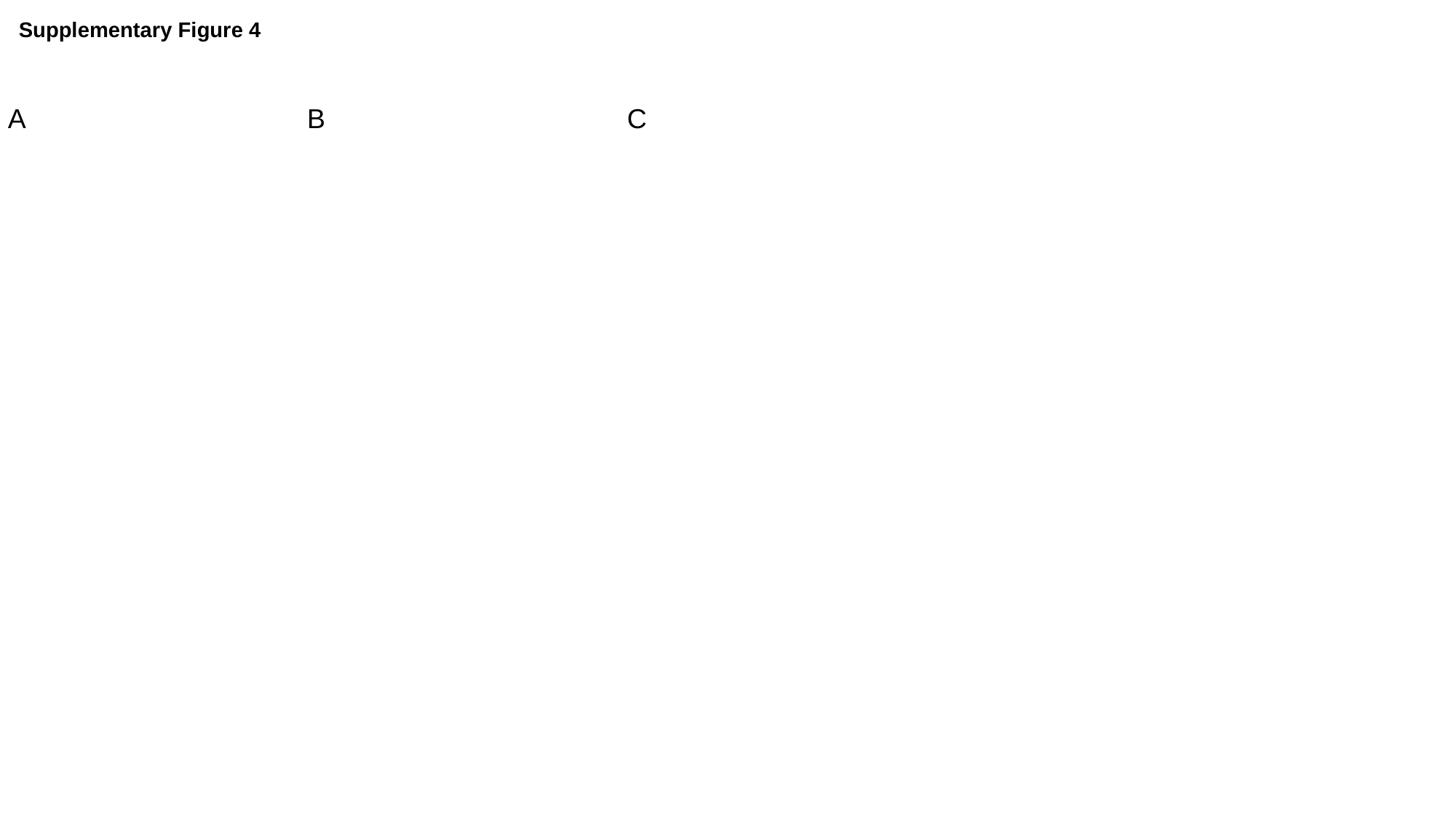

Supplementary Figure 4
A
B
C

## Slide 5
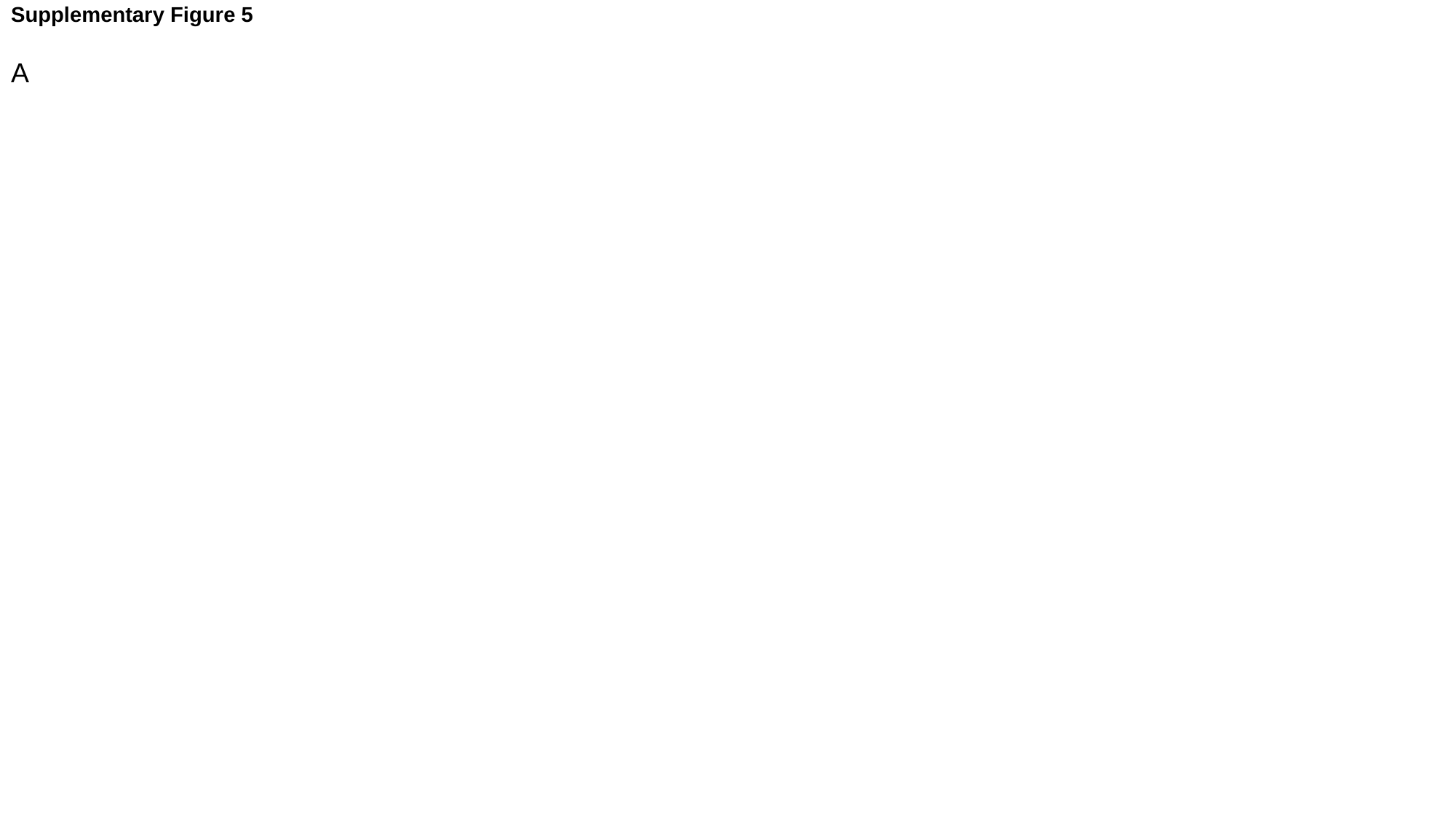

Supplementary Figure 5
A

## Slide 6
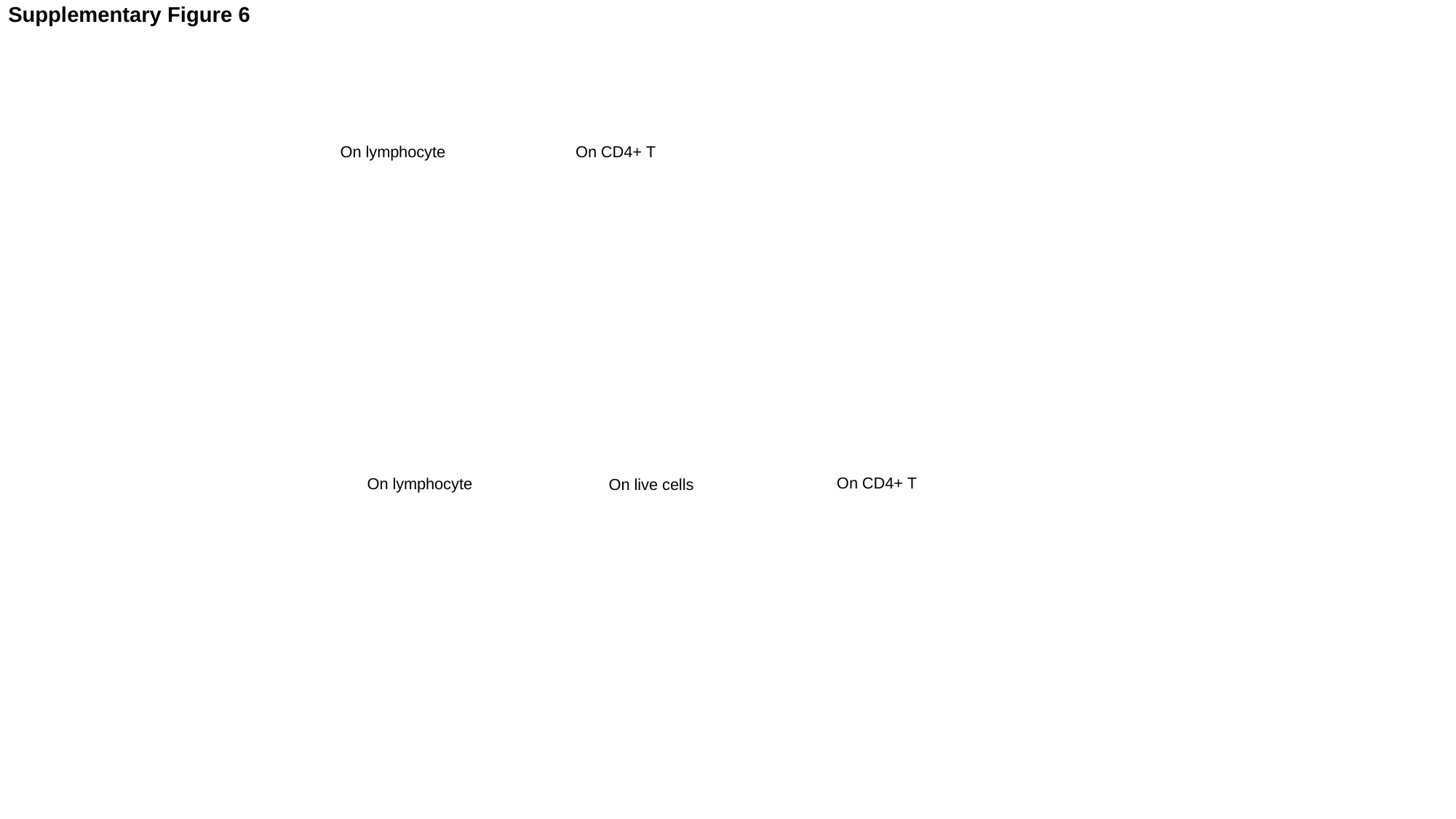

Supplementary Figure 6
On lymphocyte
On CD4+ T
On CD4+ T
On lymphocyte
On live cells
