# Supplementary material for: CD4+FoxP3+ T regulatory cells subsets release small extracellular vesicles containing cell death-related proteins as potential mechanism of T cell suppression
Source: Front Immunol. 2026 Apr 21;17:1777669. doi: 10.3389/fimmu.2026.1777669 (PMC13139319; doi:10.3389/fimmu.2026.1777669)
Supplement: Supplementary file 2 [file Table1.docx]

Table I. Exclusive proteins found on sEV obtained from nTregs

| **Gene name** | **Uniprot ID** |
| --- | --- |
| Keratin, type I cuticular Ha6 | B1AQ75 |
| Synaptonemal complex central element protein 3 | B5KM66 |
| Nucleoprotein TPR | F6ZDS4 |
| Proteasome subunit beta type-1 | O09061 |
| Ig lambda-1 chain V region H2020 | P01726 |
| Ig lambda-1 chain C region | P01843 |
| Ig gamma-1 chain C region, membrane-bound form | P01869 |
| Immunoglobulin heavy constant mu | P01872 |
| Major urinary protein 11 | P04938 |
| Tubulin alpha-1B chain | P05213 |
| Transthyretin | P07309 |
| Histone H2B type 1-F/J/L | P10853 |
| Collagen alpha-1 chain | P28481 |
| CD9 antigen | P40240 |
| Phospholipid transfer protein | P55065 |
| Nicastrin | P57716 |
| Poly-binding protein 3 | P57722 |
| Tropomyosin alpha-1 chain | P58771 |
| High mobility group protein B1 | P63158 |
| ATPase family gene 2 protein homolog A | Q3UMC0 |
| Gasdermin-A3 | Q5Y4Y6 |
| DNA- endonuclease 2 | Q68G58 |
| Beta-actin-like protein 2 | Q8BFZ3 |
| Secreted phosphoprotein 24 | Q8K1I3 |
| Hornerin | Q8VHD8 |
| Keratin, type II cuticular Hb4 | Q99M73 |
| Glutamyl-tRNA amidotransferase subunit A, mitochondrial | Q9CZN8 |
| Protein S100-A14 | Q9D2Q8 |
| Troponin T, fast skeletal muscle | Q9QZ47 |
| Hepatocyte growth factor activator | Q9R098 |

Table II. Exclusive proteins found on sEV obtained from iTregs

| Gene name | Uniprot ID |
| --- | --- |
| G patch domain-containing protein 8 | A2A6A1 |
| Equilibrative nucleobase transporter 1 | A2AVZ9 |
| Adenylyltransferase and sulfurtransferase MOCS3 | A2BDX3 |
| Acetyl-CoA carboxylase 2 | E9Q4Z2 |
| Rap1 GTPase-GDP dissociation stimulator 1 | E9Q912 |
| Voltage-dependent calcium channel subunit alpha-2/delta-1 | O08532 |
| Clathrin light chain A | O08585 |
| Synaptosomal-associated protein 23 | O09044 |
| Histone deacetylase 1 | O09106 |
| Synaptophysin-like protein 1 | O09117 |
| Tumor necrosis factor ligand superfamily member 11 | O35235 |
| Acidic leucine-rich nuclear phosphoprotein 32 family member A | O35381 |
| Neurogenic locus notch homolog protein 2 | O35516 |
| NPC intracellular cholesterol transporter 1 | O35604 |
| Secretory carrier-associated membrane protein 3 | O35609 |
| AP-1 complex subunit beta-1 | O35643 |
| NKG2-D type II integral membrane protein | O54709 |
| Pro-interleukin-16 | O54824 |
| Eukaryotic translation initiation factor 6 | O55135 |
| Eukaryotic translation initiation factor 3 subunit D | O70194 |
| Potassium/sodium hyperpolarization-activated cyclic nucleotide-gated channel 3 | O88705 |
| GRB2-related adaptor protein 2 | O89100 |
| Caspase-8 | O89110 |
| cAMP-dependent protein kinase catalytic subunit alpha | P05132 |
| Aspartate aminotransferase, cytoplasmic | P05201 |
| Proto-oncogene tyrosine-protein kinase Src | P05480 |
| Adenine phosphoribosyltransferase | P08030 |
| Tyrosine-protein kinase HCK | P08103 |
| Granzyme C | P08882 |
| Nucleolin | P09405 |
| Myosin light chain 4 | P09541 |
| Lymphocyte antigen 6C1 | P0CW02 |
| T-cell surface glycoprotein CD8 beta chain | P10300 |
| Eukaryotic initiation factor 4A-II | P10630 |
| Perforin-1 | P10820 |
| Ras-related protein R-Ras | P10833 |
| Cyclin-dependent kinase 1 | P11440 |
| T-cell surface glycoprotein CD3 gamma chain | P11942 |
| ATP-dependent 6-phosphofructokinase, liver type | P12382 |
| Myosin-3 | P13541 |
| Calreticulin | P14211 |
| Galectin-1 | P16045 |
| Spectrin alpha chain, non-erythrocytic 1 | P16546 |
| AP-2 complex subunit alpha-1 | P17426 |
| Receptor-type tyrosine-protein phosphatase alpha | P18052 |
| Protein kinase C alpha type | P20444 |
| Protein EVI2A | P20934 |
| Lactadherin | P21956 |
| Peptidyl-prolyl cis-trans isomerase B | P24369 |
| Leukotriene A-4 hydrolase | P24527 |
| Inosine-5'-monophosphate dehydrogenase 2 | P24547 |
| Tumor necrosis factor receptor superfamily member 1B | P25119 |
| Glutamate dehydrogenase 1, mitochondrial | P26443 |
| Serine--tRNA ligase, cytoplasmic | P26638 |
| Myristoylated alanine-rich C-kinase substrate | P26645 |
| Large ribosomal subunit protein uL3 | P27659 |
| Protein disulfide-isomerase A3 | P27773 |
| Cytoplasmic aconitate hydratase | P28271 |
| Nucleosome assembly protein 1-like 1 | P28656 |
| Tyrosine-protein phosphatase non-receptor type 6 | P29351 |
| Thromboxane A2 receptor | P30987 |
| Aminoacyl tRNA synthase complex-interacting multifunctional protein 1 | P31230 |
| Developmentally-regulated GTP-binding protein 1 | P32233 |
| GTPase KRas | P32883 |
| Cytokine receptor common subunit gamma | P34902 |
| Large ribosomal subunit protein eL18 | P35980 |
| Dynamin-2 | P39054 |
| Tyrosine-protein kinase Fyn | P39688 |
| Septin-1 | P42209 |
| Integrin alpha-V | P43406 |
| Ras-related protein Rab-11B | P46638 |
| Merlin | P46662 |
| Tumor necrosis factor receptor superfamily member 4 | P47741 |
| C-C chemokine receptor type 7 | P47774 |
| Large ribosomal subunit protein eL6 | P47911 |
| Large ribosomal subunit protein eL13 | P47963 |
| Fibromodulin | P50608 |
| Long-chain specific acyl-CoA dehydrogenase, mitochondrial | P51174 |
| Lumican | P51885 |
| Large ribosomal subunit protein uL1 | P53026 |
| Ras-related protein Rab-8A | P55258 |
| Cathepsin W | P56203 |
| RuvB-like 1 | P60122 |
| DnaJ homolog subfamily C member 5 | P60904 |
| Ras-related protein Rab-5B | P61021 |
| 14-3-3 protein gamma | P61982 |
| Ras-related protein R-Ras2 | P62071 |
| Serine/threonine-protein phosphatase PP1-beta catalytic subunit | P62141 |
| 26S proteasome regulatory subunit 4 | P62192 |
| Small ribosomal subunit protein uS11 | P62264 |
| Small ribosomal subunit protein eS4 | P62702 |
| Small ribosomal subunit protein eS26 | P62855 |
| Large ribosomal subunit protein eL30 | P62889 |
| V-type proton ATPase 16 kDa proteolipid subunit c | P63082 |
| Guanine nucleotide-binding protein G/G/G subunit gamma-2 | P63213 |
| 14-3-3 protein eta | P68510 |
| Na/H exchange regulatory cofactor NHE-RF1 | P70441 |
| AP-2 complex subunit mu | P84091 |
| Sodium/potassium-transporting ATPase subunit beta-3 | P97370 |
| Myosin regulatory light chain 11 | P97457 |
| Fumarate hydratase, mitochondrial | P97807 |
| FXYD domain-containing ion transport regulator 5 | P97808 |
| Glucose-6-phosphate 1-dehydrogenase X | Q00612 |
| Nucleoside diphosphate kinase B | Q01768 |
| Transitional endoplasmic reticulum ATPase | Q01853 |
| T-lymphocyte surface antigen Ly-9 | Q01965 |
| Programmed cell death protein 1 | Q02242 |
| ATP synthase subunit alpha, mitochondrial | Q03265 |
| Prefoldin subunit 6 | Q03958 |
| DNA topoisomerase 1 | Q04750 |
| Coagulation factor VIII | Q06194 |
| Glia-derived nexin | Q07235 |
| Platelet glycoprotein 4 | Q08857 |
| T-cell-specific guanine nucleotide triphosphate-binding protein 2 | Q3T9E4 |
| Ectonucleotide pyrophosphatase/phosphodiesterase family member 7 | Q3TIW9 |
| E3 ubiquitin-protein ligase CBL-B | Q3TTA7 |
| Far upstream element-binding protein 2 | Q3U0V1 |
| T-cell surface protein tactile | Q3U0X8 |
| Extended synaptotagmin-1 | Q3U7R1 |
| Protein Niban 1 | Q3UW53 |
| C2 domain-containing protein 3 | Q52KB6 |
| GTP-binding protein Di-Ras2 | Q5PR73 |
| Phosphoribosylformylglycinamidine synthase | Q5SUR0 |
| Growth factor receptor-bound protein 2 | Q60631 |
| Flotillin-2 | Q60634 |
| Immunity-related GTPase family M protein 1 | Q60766 |
| Lymphocyte antigen 75 | Q60767 |
| Syntaxin-binding protein 3 | Q60770 |
| Histone-binding protein RBBP4 | Q60972 |
| Scavenger receptor class B member 1 | Q61009 |
| NADPH oxidase 2 | Q61093 |
| Rho guanine nucleotide exchange factor 1 | Q61210 |
| Beta-2-syntrophin | Q61235 |
| B-cell receptor-associated protein 31 | Q61335 |
| Mitogen-activated protein kinase 6 | Q61532 |
| ATP-dependent RNA helicase DDX19A | Q61655 |
| Probable ATP-dependent RNA helicase DDX5 | Q61656 |
| Heat shock protein 105 kDa | Q61699 |
| Lymphocyte activation gene 3 protein | Q61790 |
| ATP-dependent RNA helicase DDX3X | Q62167 |
| Cystatin-B | Q62426 |
| Protein NDRG1 | Q62433 |
| Dual specificity mitogen-activated protein kinase kinase 2 | Q63932 |
| Coiled-coil domain-containing protein 18 | Q640L5 |
| Regucalcin | Q64374 |
| Potassium-transporting ATPase alpha chain 1 | Q64436 |
| Methionine--tRNA ligase, cytoplasmic | Q68FL6 |
| Nestin | Q6P5H2 |
| Anoctamin-6 | Q6P9J9 |
| Choline transporter-like protein 1 | Q6X893 |
| Small ribosomal subunit protein eS27 | Q6ZWU9 |
| Staphylococcal nuclease domain-containing protein 1 | Q78PY7 |
| Transmembrane channel-like protein 8 | Q7TN58 |
| Transmembrane channel-like protein 6 | Q7TN60 |
| Protein strawberry notch homolog 2 | Q7TNB8 |
| Echinoderm microtubule-associated protein-like 2 | Q7TNG5 |
| Interferon-induced very large GTPase 1 | Q80SU7 |
| Ovarian cancer G-protein coupled receptor 1 | Q8BFQ3 |
| Transportin-1 | Q8BFY9 |
| SLAM family member 7 | Q8BHK6 |
| RUN and FYVE domain-containing protein 1 | Q8BIJ7 |
| Isoleucine--tRNA ligase, cytoplasmic | Q8BU30 |
| Ras-related protein Rap-2c | Q8BU31 |
| Low-density lipoprotein receptor class A domain-containing protein 4 | Q8BWJ4 |
| Serine/threonine-protein kinase TAO3 | Q8BYC6 |
| Rho-related GTP-binding protein RhoF | Q8BYP3 |
| Leucyl-cystinyl aminopeptidase | Q8C129 |
| Copine-1 | Q8C166 |
| RAS protein activator like-3 | Q8C2K5 |
| Dedicator of cytokinesis protein 2 | Q8C3J5 |
| Signaling threshold-regulating transmembrane adapter 1 | Q8C503 |
| E3 ubiquitin-protein ligase Itchy | Q8C863 |
| Sodium-coupled neutral amino acid symporter 2 | Q8CFE6 |
| DNA-directed RNA polymerase II subunit RPB2 | Q8CFI7 |
| Calcium homeostasis endoplasmic reticulum protein | Q8CGZ0 |
| Metalloreductase STEAP3 | Q8CI59 |
| Serine/threonine-protein kinase PAK 2 | Q8CIN4 |
| Eukaryotic translation initiation factor 3 subunit B | Q8JZQ9 |
| Arf-GAP with coiled-coil, ANK repeat and PH domain-containing protein 1 | Q8K2H4 |
| BRO1 domain-containing protein BROX | Q8K2Q7 |
| L-threonine 3-dehydrogenase, mitochondrial | Q8K3F7 |
| Eukaryotic peptide chain release factor GTP-binding subunit ERF3A | Q8R050 |
| Heterogeneous nuclear ribonucleoprotein L | Q8R081 |
| Vacuolar protein sorting-associated protein 37C | Q8R105 |
| Aquaporin-3 | Q8R2N1 |
| Urocanate hydratase | Q8VC12 |
| Delta-sterol reductase | Q8VCH6 |
| Palmitoyltransferase ZDHHC5 | Q8VDZ4 |
| Programmed cell death protein 10 | Q8VE70 |
| Protein phosphatase 1 regulatory inhibitor subunit 16B | Q8VHQ3 |
| Sorting nexin-9 | Q91VH2 |
| Protein MEMO1 | Q91VH6 |
| eIF5-mimic protein 1 | Q91VK1 |
| Metal cation symporter ZIP8 | Q91W10 |
| N-acetylmannosamine kinase | Q91WG8 |
| Brain acid soluble protein 1 | Q91XV3 |
| Beta-arrestin-2 | Q91YI4 |
| SLIT-ROBO Rho GTPase-activating protein 2 | Q91Z67 |
| Sorting nexin-18 | Q91ZR2 |
| Prolow-density lipoprotein receptor-related protein 1 | Q91ZX7 |
| Tubulin beta-6 chain | Q922F4 |
| Protein disulfide-isomerase A6 | Q922R8 |
| Transaldolase | Q93092 |
| Syntenin-2 | Q99JZ0 |
| NAD-dependent malic enzyme, mitochondrial | Q99KE1 |
| Nicotinamide phosphoribosyltransferase | Q99KQ4 |
| Charged multivesicular body protein 1B1 | Q99LU0 |
| Lysine--tRNA ligase | Q99MN1 |
| Interferon-induced transmembrane protein 3 | Q9CQW9 |
| Vacuolar protein sorting-associated protein VTA1 homolog | Q9CR26 |
| Ribose-phosphate pyrophosphokinase 2 | Q9CS42 |
| Small ribosomal subunit protein eS19 | Q9CZX8 |
| Heterogeneous nuclear ribonucleoprotein M | Q9D0E1 |
| Interferon-induced transmembrane protein 1 | Q9D103 |
| Proton-activated chloride channel | Q9D771 |
| Inorganic pyrophosphatase | Q9D819 |
| Large ribosomal subunit protein uL4 | Q9D8E6 |
| 26S proteasome non-ATPase regulatory subunit 12 | Q9D8W5 |
| Phosphoglycerate mutase 1 | Q9DBJ1 |
| Eukaryotic translation initiation factor 3 subunit F | Q9DCH4 |
| Lck-interacting transmembrane adapter 1 | Q9EQR5 |
| Secretory carrier-associated membrane protein 2 | Q9ERN0 |
| Beta-parvin | Q9ES46 |
| Tripartite motif-containing protein 2 | Q9ESN6 |
| H/ACA ribonucleoprotein complex subunit DKC1 | Q9ESX5 |
| Glycogen phosphorylase, liver form | Q9ET01 |
| SLAM family member 6 | Q9ET39 |
| Tropomodulin-3 | Q9JHJ0 |
| Inositol-3-phosphate synthase 1 | Q9JHU9 |
| Formin-like protein 1 | Q9JL26 |
| Protein PALS2 | Q9JLB0 |
| 4-trimethylaminobutyraldehyde dehydrogenase | Q9JLJ2 |
| Hypermethylated in cancer 2 protein | Q9JLZ6 |
| Single Ig IL-1-related receptor | Q9JLZ8 |
| Misshapen-like kinase 1 | Q9JM52 |
| Integrin alpha-IIb | Q9QUM0 |
| Prolyl endopeptidase | Q9QUR6 |
| EH domain-containing protein 3 | Q9QXY6 |
| Core histone macro-H2A.1 | Q9QZQ8 |
| Four and a half LIM domains protein 3 | Q9R059 |
| Destrin | Q9R0P5 |
| Proteasome subunit alpha type-4 | Q9R1P0 |
| Septin-6 | Q9R1T4 |
| Transient receptor potential cation channel subfamily V member 2 | Q9WTR1 |
| Coronin-1C | Q9WUM4 |
| Serine/threonine-protein kinase TBK1 | Q9WUN2 |
| Protein kinase C and casein kinase substrate in neurons protein 2 | Q9WVE8 |
| Guanylate-binding protein 2 | Q9Z0E6 |
| CUGBP Elav-like family member 2 | Q9Z0H4 |
| Eukaryotic translation initiation factor 2 subunit 3, X-linked | Q9Z0N1 |
| Spliceosome RNA helicase Ddx39b | Q9Z1N5 |
| Keratin, type II cuticular Hb5 | Q9Z2T6 |
| Heterogeneous nuclear ribonucleoprotein F | Q9Z2X1 |

Table III. Exclusive proteins found on sEV obtained from RATregs

| Gene name | Uniprot ID |
| --- | --- |
| Peroxiredoxin-6 | O08709 |
| Prohibitin-2 | O35129 |
| 5'-3' exonuclease PLD3 | O35405 |
| 26S proteasome non-ATPase regulatory subunit 14 | O35593 |
| Dolichyl-diphosphooligosaccharide--protein glycosyltransferase 48 kDa subunit | O54734 |
| OX-2 membrane glycoprotein | O54901 |
| Barrier-to-autointegration factor | O54962 |
| Proteasome subunit beta type-5 | O55234 |
| Elongation factor 1-beta | O70251 |
| Band 4.1-like protein 2 | O70318 |
| General transcription factor IIH subunit 4 | O70422 |
| Proteasome subunit alpha type-3 | O70435 |
| C-X-C chemokine receptor type 3 | O88410 |
| Caspase-8 | O89110 |
| Fructose-bisphosphate aldolase C | P05063 |
| ATP-dependent translocase ABCB1 | P06795 |
| Superoxide dismutase | P08228 |
| GTPase NRas | P08556 |
| Charged multivesicular body protein 6 | P0C0A3 |
| Histone H2A.Z | P0C0S6 |
| Nidogen-1 | P10493 |
| Histone H2B type 1-M | P10854 |
| Collagen alpha-1 chain | P11087 |
| Uridine 5'-monophosphate synthase | P13439 |
| Glutathione S-transferase A1 | P13745 |
| Nucleoside diphosphate kinase A | P15532 |
| Argininosuccinate synthase | P16460 |
| Vitamin D-binding protein | P21614 |
| Peptidyl-prolyl cis-trans isomerase FKBP1A | P26883 |
| Protein disulfide-isomerase A3 | P27773 |
| CUGBP Elav-like family member 1 | P28659 |
| Meprin A subunit alpha | P28825 |
| Ferritin light chain 1 | P29391 |
| Chromosome-associated kinesin KIF4 | P33174 |
| Early activation antigen CD69 | P37217 |
| Histone H1.3 | P43277 |
| F-actin-capping protein subunit alpha-1 | P47753 |
| Insulin-like growth factor-binding protein 2 | P47877 |
| Large ribosomal subunit protein eL13 | P47963 |
| Elongation factor 1-delta | P57776 |
| RuvB-like 1 | P60122 |
| Eukaryotic translation initiation factor 3 subunit E | P60229 |
| Ras-related protein Rap-2b | P61226 |
| Ras-related protein R-Ras2 | P62071 |
| Small ribosomal subunit protein eS7 | P62082 |
| 26S proteasome regulatory subunit 8 | P62196 |
| Small ribosomal subunit protein uS12 | P62267 |
| 26S proteasome regulatory subunit 10B | P62334 |
| Ras-related protein Rab-11A | P62492 |
| Myotrophin | P62774 |
| Small ribosomal subunit protein eS24 | P62849 |
| Vesicle-associated membrane protein 3 | P63024 |
| Mitogen-activated protein kinase 1 | P63085 |
| Tubulin alpha-1A chain | P68369 |
| Ecto-ADP-ribosyltransferase 5 | P70352 |
| Four and a half LIM domains protein 1 | P97447 |
| Peroxiredoxin-5, mitochondrial | P99029 |
| Integrin alpha-4 | Q00651 |
| Collagen alpha-2 chain | Q01149 |
| Collagen alpha-2 chain | Q02788 |
| Protein AMBP ; Inter-alpha-trypsin inhibitor light chain | Q07456 |
| Serine/threonine-protein kinase PLK1 | Q07832 |
| Calponin-2 | Q08093 |
| Sodium-coupled neutral amino acid transporter 5 | Q3U1J0 |
| Bromodomain-containing protein 9 | Q3UQU0 |
| GTP-binding protein Di-Ras2 | Q5PR73 |
| Myosin light polypeptide 6 | Q60605 |
| Heterogeneous nuclear ribonucleoprotein D0 | Q60668 |
| Deoxynucleoside triphosphate triphosphohydrolase SAMHD1 | Q60710 |
| Casein kinase II subunit alpha | Q60737 |
| Tumor necrosis factor alpha-induced protein 3 | Q60769 |
| Non-selective voltage-gated ion channel VDAC1 | Q60932 |
| Ceruloplasmin | Q61147 |
| Platelet-activating factor acetylhydrolase IB subunit alpha2 | Q61206 |
| Heat shock 70 kDa protein 4 | Q61316 |
| D-3-phosphoglycerate dehydrogenase | Q61753 |
| DNA replication licensing factor MCM7 | Q61881 |
| Nucleophosmin | Q61937 |
| Spectrin beta chain, non-erythrocytic 1 | Q62261 |
| Drebrin-like protein | Q62418 |
| 10 kDa heat shock protein, mitochondrial | Q64433 |
| Tripeptidyl-peptidase 2 | Q64514 |
| Myoferlin | Q69ZN7 |
| Tropomyosin alpha-4 chain | Q6IRU2 |
| Sorcin | Q6P069 |
| U5 small nuclear ribonucleoprotein 200 kDa helicase | Q6P4T2 |
| Carboxypeptidase A4 | Q6P8K8 |
| Stomatin-like protein 3 | Q6PE84 |
| Choline transporter-like protein 1 | Q6X893 |
| Heterogeneous nuclear ribonucleoprotein Q | Q7TMK9 |
| Transmembrane channel-like protein 6 | Q7TN60 |
| Alpha-actinin-1 | Q7TPR4 |
| Epsin-1 | Q80VP1 |
| Hyaluronan and proteoglycan link protein 4 | Q80WM4 |
| Coiled-coil domain-containing protein 50 | Q810U5 |
| Elongation factor Tu, mitochondrial | Q8BFR5 |
| Peflin | Q8BFY6 |
| Ubiquitin-associated and SH3 domain-containing protein B | Q8BGG7 |
| CDC42 small effector protein 2 | Q8BGH7 |
| Y+L amino acid transporter 2 | Q8BGK6 |
| Complement component C8 beta chain | Q8BH35 |
| FERM domain-containing protein 4A | Q8BIE6 |
| Importin-5 | Q8BKC5 |
| Asparagine--tRNA ligase, cytoplasmic | Q8BP47 |
| GTPase IMAP family member 5 | Q8BWF2 |
| Unconventional myosin-IXa | Q8C170 |
| Transmembrane protein C16orf54 homolog | Q8C708 |
| Abl interactor 1 | Q8CBW3 |
| Fibrinogen beta chain | Q8K0E8 |
| Arf-GAP with coiled-coil, ANK repeat and PH domain-containing protein 1 | Q8K2H4 |
| Bleomycin hydrolase | Q8R016 |
| Splicing factor, proline- and glutamine-rich | Q8VIJ6 |
| KICSTOR complex protein ITFG2 | Q91WI7 |
| L-xylulose reductase | Q91X52 |
| C-1-tetrahydrofolate synthase, cytoplasmic | Q922D8 |
| Flavin reductase | Q923D2 |
| Heterogeneous nuclear ribonucleoprotein A/B | Q99020 |
| N-acetylneuraminate-9-phosphate synthase | Q99J77 |
| PC4 and SFRS1-interacting protein | Q99JF8 |
| Aconitate hydratase, mitochondrial | Q99KI0 |
| Dipeptidyl peptidase 3 | Q99KK7 |
| Hsc70-interacting protein | Q99L47 |
| Actin-related protein 2/3 complex subunit 5 | Q9CPW4 |
| 6-phosphogluconolactonase | Q9CQ60 |
| Peptidyl-prolyl cis-trans isomerase D | Q9CR16 |
| Prefoldin subunit 1 | Q9CWM4 |
| Citrate synthase | Q9CZU6 |
| Acyl-CoA-binding domain-containing protein 7 | Q9D258 |
| Coronin-7 | Q9D2V7 |
| Rho-related GTP-binding protein RhoH | Q9D3G9 |
| Ribose-phosphate pyrophosphokinase 1 | Q9D7G0 |
| Charged multivesicular body protein 5 | Q9D7S9 |
| Large ribosomal subunit protein uL4 | Q9D8E6 |
| Eukaryotic translation initiation factor 3 subunit K | Q9DBZ5 |
| Exportin-2 | Q9ERK4 |
| Pleckstrin | Q9JHK5 |
| Phospholipid scramblase 3 | Q9JIZ9 |
| Proteasome subunit alpha type-6 | Q9QUM9 |
| Protein NDRG3 | Q9QYF9 |
| Cartilage oligomeric matrix protein | Q9R0G6 |
| Proteasome subunit beta type-3 | Q9R1P1 |
| Proteolipid protein 2 | Q9R1Q7 |
| SUMO-activating enzyme subunit 1 | Q9R1T2 |
| Coronin-1C | Q9WUM4 |
| Eukaryotic translation initiation factor 3 subunit G | Q9Z1D1 |
| Proteasome subunit alpha type-5 | Q9Z2U1 |

Table IV. Proteins found on sEV obtained from iTregs and RATregs

| Gene name | Uniprot ID |
| --- | --- |
| Centriolin | A2AL36 |
| Tubulin beta-1 chain | A2AQ07 |
| Adenylyltransferase and sulfurtransferase MOCS3 | A2BDX3 |
| Immunoglobulin superfamily member 2 | A8E0Y8 |
| Multifunctional protein CAD | B2RQC6 |
| Phosphatidylinositol 4-kinase alpha | E9Q3L2 |
| Plasma membrane calcium-transporting ATPase 1 | G5E829 |
| Dihydropyrimidinase-related protein 2 | O08553 |
| Flotillin-1 | O08917 |
| Sodium- and chloride-dependent taurine transporter | O35316 |
| Calpain-1 catalytic subunit | O35350 |
| Disintegrin and metalloproteinase domain-containing protein 10 | O35598 |
| Tumor necrosis factor receptor superfamily member 18 | O35714 |
| Heterogeneous nuclear ribonucleoprotein H | O35737 |
| Integrin beta-3 | O54890 |
| Linker for activation of T-cells family member 1 | O54957 |
| Serine/threonine-protein kinase 10 | O55098 |
| Septin-7 | O55131 |
| Scaffold protein ILK | O55222 |
| ATP-dependent RNA helicase A | O70133 |
| WD repeat-containing protein 1 | O88342 |
| Heterogeneous nuclear ribonucleoproteins A2/B1 | O88569 |
| Coagulation factor V | O88783 |
| Coagulation factor X | O88947 |
| Alpha-amylase 1 | P00687 |
| Complement C4-B | P01029 |
| T-cell receptor alpha chain constant | P01849 |
| H-2 class I histocompatibility antigen, Q10 alpha chain | P01898 |
| H-2 class I histocompatibility antigen, D-B alpha chain | P01899 |
| H-2 class I histocompatibility antigen, K-B alpha chain | P01901 |
| Granzyme B | P04187 |
| Aspartate aminotransferase, mitochondrial | P05202 |
| Lymphocyte antigen 6A-2/6E-1 | P05533 |
| Proto-oncogene tyrosine-protein kinase LCK | P06240 |
| T-cell surface glycoprotein CD4 | P06332 |
| Complement C5 | P06684 |
| Ribonucleoside-diphosphate reductase large subunit | P07742 |
| Endoplasmin | P08113 |
| T-cell surface antigen CD2 | P08920 |
| Integrin beta-1 | P09055 |
| Calmodulin-2 | P0DP27 |
| T-cell surface glycoprotein CD8 beta chain | P10300 |
| MLV-related proviral Env polyprotein | P10404 |
| Thioredoxin | P10639 |
| Granzyme A | P11032 |
| T-complex protein 1 subunit alpha | P11983 |
| Programmed cell death protein 6 | P12815 |
| Large ribosomal subunit protein eL8 | P12970 |
| Intercellular adhesion molecule 1 | P13597 |
| Serglycin | P13609 |
| Sodium/potassium-transporting ATPase subunit beta-1 | P14094 |
| Large ribosomal subunit protein uL15 | P14115 |
| Small ribosomal subunit protein uS9 | P14131 |
| Large ribosomal subunit protein uL30 | P14148 |
| Small ribosomal subunit protein uS2 | P14206 |
| Calreticulin | P14211 |
| H-2 class I histocompatibility antigen, Q7 alpha chain | P14429 |
| H-2 class II histocompatibility antigen, A-B alpha chain | P14434 |
| 26S proteasome non-ATPase regulatory subunit 3 | P14685 |
| Large ribosomal subunit protein uL10 | P14869 |
| Carbonic anhydrase 3 | P16015 |
| 2',3'-cyclic-nucleotide 3'-phosphodiesterase | P16330 |
| Polypyrimidine tract-binding protein 1 | P17225 |
| Solute carrier family 2, facilitated glucose transporter member 1 | P17809 |
| Proliferating cell nuclear antigen | P17918 |
| CD48 antigen | P18181 |
| Ig heavy chain V region 5-84 | P18525 |
| Basigin | P18572 |
| Fatty acid synthase | P19096 |
| Glutathione S-transferase P 1 | P19157 |
| Vimentin | P20152 |
| Tropomyosin alpha-3 chain | P21107 |
| T-cell surface glycoprotein CD3 epsilon chain | P22646 |
| Eukaryotic translation initiation factor 3 subunit A | P23116 |
| Purine nucleoside phosphorylase | P23492 |
| T-cell surface glycoprotein CD3 zeta chain | P24161 |
| Cation-dependent mannose-6-phosphate receptor | P24668 |
| Small ribosomal subunit protein uS5 | P25444 |
| Integrin beta-7 | P26011 |
| Talin-1 | P26039 |
| Ezrin | P26040 |
| Plasma kallikrein | P26262 |
| 26S proteasome non-ATPase regulatory subunit 7 | P26516 |
| Guanine nucleotide-binding protein subunit alpha-13 | P27601 |
| Large ribosomal subunit protein uL3 | P27659 |
| Histone H2AX | P27661 |
| Dipeptidyl peptidase 4 | P28843 |
| Polyadenylate-binding protein 1 | P29341 |
| Alpha-2-HS-glycoprotein | P29699 |
| Ras-related protein Rab-5C | P35278 |
| Ras-related protein Rab-21 | P35282 |
| CD81 antigen | P35762 |
| Large ribosomal subunit protein uL11 | P35979 |
| Tyrosine-protein kinase CSK | P41241 |
| T-complex protein 1 subunit theta | P42932 |
| Histone H1.4 | P43274 |
| Tyrosine-protein kinase ZAP-70 | P43404 |
| Vacuolar protein sorting-associated protein 4B | P46467 |
| 26S proteasome regulatory subunit 7 | P46471 |
| E3 ubiquitin-protein ligase NEDD4 | P46935 |
| F-actin-capping protein subunit alpha-2 | P47754 |
| Large ribosomal subunit protein uL18 | P47962 |
| Citron Rho-interacting kinase | P49025 |
| Heparin cofactor 2 | P49182 |
| Hematopoietic lineage cell-specific protein | P49710 |
| V-type proton ATPase catalytic subunit A | P50516 |
| Ras-related protein Rab-7a | P51150 |
| Large ribosomal subunit protein uL6 | P51410 |
| ADP/ATP translocase 2 | P51881 |
| Monocarboxylate transporter 1 | P53986 |
| Stomatin | P54116 |
| Adenylosuccinate lyase | P54822 |
| Ectonucleoside triphosphate diphosphohydrolase 1 | P55772 |
| ATP synthase subunit beta, mitochondrial | P56480 |
| ADP-ribosyl cyclase/cyclic ADP-ribose hydrolase 1 | P56528 |
| Monocarboxylate transporter 4 | P57787 |
| TBC1 domain family member 10A | P58802 |
| Actin-related protein 2/3 complex subunit 4 | P59999 |
| Poly-binding protein 1 | P60335 |
| Cell division control protein 42 homolog | P60766 |
| Ras-related protein Rab-10 | P61027 |
| Ras-related protein Rab-8B | P61028 |
| Actin-related protein 2 | P61161 |
| ADP-ribosylation factor 3 | P61205 |
| Large ribosomal subunit protein uL24 | P61255 |
| Proteasome activator complex subunit 3 | P61290 |
| Serine/threonine-protein phosphatase PP1-alpha catalytic subunit | P62137 |
| Small ribosomal subunit protein uS13 | P62270 |
| ADP-ribosylation factor 6 | P62331 |
| Small ribosomal subunit protein eS4 | P62702 |
| Serine/threonine-protein phosphatase 2A catalytic subunit beta isoform | P62715 |
| Large ribosomal subunit protein eL20 | P62717 |
| Ras-related protein Rab-1A | P62821 |
| GTP-binding nuclear protein Ran | P62827 |
| Ras-related protein Rap-1A | P62835 |
| Small ribosomal subunit protein eS25 | P62852 |
| Guanine nucleotide-binding protein G/G/G subunit beta-1 | P62874 |
| Small ribosomal subunit protein uS3 | P62908 |
| Large ribosomal subunit protein uL2 | P62918 |
| Y-box-binding protein 1 | P62960 |
| Ras-related C3 botulinum toxin substrate 1 | P63001 |
| Translationally-controlled tumor protein | P63028 |
| DnaJ homolog subfamily A member 1 | P63037 |
| 60 kDa heat shock protein, mitochondrial | P63038 |
| Guanine nucleotide-binding protein G subunit alpha isoforms short | P63094 |
| 14-3-3 protein zeta/delta | P63101 |
| Casein kinase II subunit beta | P67871 |
| Small ribosomal subunit protein RACK1 | P68040 |
| Tubulin alpha-4A chain | P68368 |
| Importin subunit beta-1 | P70168 |
| Phosphatidylethanolamine-binding protein 1 | P70296 |
| Na/H exchange regulatory cofactor NHE-RF1 | P70441 |
| Syntaxin-4 | P70452 |
| Vasodilator-stimulated phosphoprotein | P70460 |
| T-complex protein 1 subunit eta | P80313 |
| T-complex protein 1 subunit beta | P80314 |
| T-complex protein 1 subunit delta | P80315 |
| T-complex protein 1 subunit epsilon | P80316 |
| T-complex protein 1 subunit zeta | P80317 |
| T-complex protein 1 subunit gamma | P80318 |
| Traf2 and NCK-interacting protein kinase | P83510 |
| Rho-related GTP-binding protein RhoG | P84096 |
| Large ribosomal subunit protein uL16-like | P86048 |
| DNA replication licensing factor MCM2 | P97310 |
| Neuropilin-1 | P97333 |
| Small ribosomal subunit protein eS1 | P97351 |
| Sodium/potassium-transporting ATPase subunit beta-3 | P97370 |
| Annexin A4 | P97429 |
| Mitogen-activated protein kinase kinase kinase kinase 4 | P97820 |
| DNA topoisomerase 2-alpha | Q01320 |
| Guanylate-binding protein 1 | Q01514 |
| Neurogenic locus notch homolog protein 1 | Q01705 |
| Collagen alpha-1 chain | Q04857 |
| Ras-related C3 botulinum toxin substrate 2 | Q05144 |
| Annexin A7 | Q07076 |
| Fibulin-1 | Q08879 |
| High affinity cationic amino acid transporter 1 | Q09143 |
| Rho GTPase-activating protein 45 | Q3TBD2 |
| 26S proteasome non-ATPase regulatory subunit 1 | Q3TXS7 |
| DNA damage-binding protein 1 | Q3U1J4 |
| Disco-interacting protein 2 homolog B | Q3UH60 |
| Protein CDV3 | Q4VAA2 |
| Integrin alpha-E | Q60677 |
| Immunity-related GTPase family M protein 1 | Q60766 |
| Stress-induced-phosphoprotein 1 | Q60864 |
| T-cell differentiation antigen CD6 | Q61003 |
| Tumor susceptibility gene 101 protein | Q61187 |
| Leukocyte antigen CD37 | Q61470 |
| CD166 antigen | Q61490 |
| Haptoglobin | Q61646 |
| Leukocyte surface antigen CD47 | Q61735 |
| Integrin alpha-6 | Q61739 |
| LIM and SH3 domain protein 1 | Q61792 |
| Poly-binding protein 2 | Q61990 |
| P-selectin glycoprotein ligand 1 | Q62170 |
| Transcription intermediary factor 1-beta | Q62318 |
| Syntaxin-binding protein 2 | Q64324 |
| Receptor-type tyrosine-protein phosphatase eta | Q64455 |
| Spermidine synthase | Q64674 |
| Protein tyrosine phosphatase receptor type C-associated protein | Q64697 |
| Complement component receptor 1-like protein | Q64735 |
| Histone H2A type 2-A | Q6GSS7 |
| Multivesicular body subunit 12B | Q6KAU4 |
| Protein tweety homolog 3 | Q6P5F7 |
| Ras-related protein Rab-35 | Q6PHN9 |
| Plasma membrane calcium-transporting ATPase 4 | Q6Q477 |
| Cullin-associated NEDD8-dissociated protein 1 | Q6ZQ38 |
| Serine/threonine-protein phosphatase 2A 65 kDa regulatory subunit A alpha isoform | Q76MZ3 |
| Multivesicular body subunit 12A | Q78HU3 |
| Septin-9 | Q80UG5 |
| Heterogeneous nuclear ribonucleoprotein A3 | Q8BG05 |
| 26S proteasome non-ATPase regulatory subunit 11 | Q8BG32 |
| Protein EFR3 homolog A | Q8BG67 |
| Actin-binding protein WASF2 | Q8BH43 |
| Large ribosomal subunit protein eL24 | Q8BP67 |
| Centrosomal protein of 55 kDa | Q8BT07 |
| Copine-3 | Q8BT60 |
| Dedicator of cytokinesis protein 10 | Q8BZN6 |
| Bifunctional glutamate/proline--tRNA ligase | Q8CGC7 |
| Fermitin family homolog 3 | Q8K1B8 |
| Nck-associated protein 1-like | Q8K1X4 |
| High affinity copper uptake protein 1 | Q8K211 |
| BRO1 domain-containing protein BROX | Q8K2Q7 |
| CD226 antigen | Q8K4F0 |
| Eukaryotic translation initiation factor 3 subunit L | Q8QZY1 |
| Tetraspanin-14 | Q8QZY6 |
| 26S proteasome non-ATPase regulatory subunit 2 | Q8VDM4 |
| Cell cycle control protein 50A | Q8VEK0 |
| Heterogeneous nuclear ribonucleoprotein U | Q8VEK3 |
| C-type lectin domain family 2 member D | Q91V08 |
| SH3 domain-binding glutamic acid-rich-like protein 3 | Q91VW3 |
| Eukaryotic translation initiation factor 3 subunit H | Q91WK2 |
| Splicing factor 3B subunit 3 | Q921M3 |
| CYFIP-related Rac1 interactor B | Q921M7 |
| Aspartate--tRNA ligase, cytoplasmic | Q922B2 |
| DNA dC->dU-editing enzyme APOBEC-3 | Q99J72 |
| 26S proteasome non-ATPase regulatory subunit 6 | Q99JI4 |
| Ras-related protein Rap-1b | Q99JI6 |
| GTPase IMAP family member 4 | Q99JY3 |
| Actin-related protein 3 | Q99JY9 |
| Protein regulator of cytokinesis 1 | Q99K43 |
| Phosphoserine aminotransferase | Q99K85 |
| Arrestin domain-containing protein 1 | Q99KN1 |
| RNA-splicing ligase RtcB homolog | Q99LF4 |
| Heat shock 70 kDa protein 14 | Q99M31 |
| Large ribosomal subunit protein uL22 | Q9CPR4 |
| Small ribosomal subunit protein eS21 | Q9CQR2 |
| 14-3-3 protein beta/alpha | Q9CQV8 |
| Claudin domain-containing protein 1 | Q9CQX5 |
| Large ribosomal subunit protein eL14 | Q9CR57 |
| Actin-related protein 2/3 complex subunit 2 | Q9CVB6 |
| Proteasome subunit alpha type-8 | Q9CWH6 |
| Bifunctional purine biosynthesis protein ATIC | Q9CWJ9 |
| IST1 homolog | Q9CX00 |
| Large ribosomal subunit protein uL5 | Q9CXW4 |
| CKLF-like MARVEL transmembrane domain-containing protein 6 | Q9CZ69 |
| Glycine--tRNA ligase | Q9CZD3 |
| Large ribosomal subunit protein eL15 | Q9CZM2 |
| Arginine--tRNA ligase, cytoplasmic | Q9D0I9 |
| Sushi domain-containing protein 3 | Q9D176 |
| Cytosolic non-specific dipeptidase | Q9D1A2 |
| Vacuolar protein sorting-associated protein 28 homolog | Q9D1C8 |
| Acetoacetyl-CoA synthetase | Q9D2R0 |
| Protein transport protein Sec23B | Q9D662 |
| Charged multivesicular body protein 2a | Q9DB34 |
| Phosphoglycerate mutase 1 | Q9DBJ1 |
| Guanine nucleotide-binding protein G subunit alpha-3 | Q9DC51 |
| 6-phosphogluconate dehydrogenase, decarboxylating | Q9DCD0 |
| Major vault protein | Q9EQK5 |
| EH domain-containing protein 4 | Q9EQP2 |
| Protein arginine N-methyltransferase 1 | Q9JIF0 |
| Equilibrative nucleoside transporter 1 | Q9JIM1 |
| Phospholipid scramblase 1 | Q9JJ00 |
| Carboxypeptidase N catalytic chain | Q9JJN5 |
| MYG1 exonuclease | Q9JK81 |
| Ras GTPase-activating-like protein IQGAP1 | Q9JKF1 |
| Lipopolysaccharide-induced tumor necrosis factor-alpha factor homolog | Q9JLJ0 |
| Actin-related protein 2/3 complex subunit 3 | Q9JM76 |
| Transforming protein RhoA | Q9QUI0 |
| Long-chain-fatty-acid--CoA ligase 4 | Q9QUJ7 |
| Signaling lymphocytic activation molecule | Q9QUM4 |
| Chloride intracellular channel protein 4 | Q9QYB1 |
| DnaJ homolog subfamily A member 2 | Q9QYJ0 |
| Interferon-inducible GTPase 1 | Q9QZ85 |
| Eukaryotic translation initiation factor 3 subunit I | Q9QZD9 |
| Phospholipid-transporting ATPase 11C | Q9QZW0 |
| Galactokinase | Q9R0N0 |
| Proteasome subunit alpha type-1 | Q9R1P4 |
| Unconventional myosin-Ic | Q9WTI7 |
| RuvB-like 2 | Q9WTM5 |
| ATP-dependent 6-phosphofructokinase, platelet type | Q9WUA3 |
| Coronin-1B | Q9WUM3 |
| Actin-related protein 2/3 complex subunit 1B | Q9WV32 |
| Prostaglandin F2 receptor negative regulator | Q9WV91 |
| Transgelin-2 | Q9WVA4 |
| Rac GTPase-activating protein 1 | Q9WVM1 |
| Inducible T-cell costimulator | Q9WVS0 |
| Adhesion G protein-coupled receptor E5 | Q9Z0M6 |
| Twinfilin-2 | Q9Z0P5 |
| Large neutral amino acids transporter small subunit 1 | Q9Z127 |
| Valine--tRNA ligase | Q9Z1Q9 |
| Heterogeneous nuclear ribonucleoproteins C1/C2 | Q9Z204 |

Table V. Proteins found in common on nTregs, iTregs and RATregs

| Gene name | Uniprot ID |
| --- | --- |
| Inter alpha-trypsin inhibitor, heavy chain 4 | A6X935 |
| Histone H2A type 1-G | C0HKE5 |
| Desmoplakin | E9Q557 |
| Kinesin-like protein KIF23 | E9Q5G3 |
| Kinesin-like protein KIF14 | L0N7N1 |
| Syntenin-1 | O08992 |
| Afamin | O89020 |
| Coronin-1A | O89053 |
| Complement C3 | P01027 |
| Interleukin-2 receptor subunit alpha | P01590 |
| T-cell surface glycoprotein CD8 alpha chain | P01731 |
| Thy-1 membrane glycoprotein | P01831 |
| T-cell receptor beta-2 chain C region | P01851 |
| Hemoglobin subunit alpha | P01942 |
| Hemoglobin subunit beta-1 | P02088 |
| Histone H3.3C | P02301 |
| Keratin, type I cytoskeletal 10 | P02535 |
| Keratin, type II cytoskeletal 1 | P04104 |
| Complement factor B | P04186 |
| Fructose-bisphosphate aldolase A | P05064 |
| Myosin light chain 1/3, skeletal muscle isoform | P05977 |
| L-lactate dehydrogenase A chain | P06151 |
| Glucose-6-phosphate isomerase | P06745 |
| Receptor-type tyrosine-protein phosphatase C | P06800 |
| Creatine kinase M-type | P07310 |
| Annexin A2 | P07356 |
| Albumin | P07724 |
| Heat shock protein HSP 90-alpha | P07901 |
| Malate dehydrogenase, mitochondrial | P08249 |
| Guanine nucleotide-binding protein G subunit alpha-2 | P08752 |
| Phosphoglycerate kinase 1 | P09411 |
| Elongation factor 1-alpha 1 | P10126 |
| Amino acid transporter heavy chain SLC3A2 | P10852 |
| Fibronectin | P11276 |
| Lysosome-associated membrane glycoprotein 1 | P11438 |
| Heat shock protein HSP 90-beta | P11499 |
| Keratin, type II cytoskeletal 8 | P11679 |
| Integrin beta-2 | P11835 |
| Gelsolin | P13020 |
| T-cell surface glycoprotein CD5 | P13379 |
| Malate dehydrogenase, cytoplasmic | P14152 |
| H-2 class II histocompatibility antigen, A beta chain | P14483 |
| Annexin A6 | P14824 |
| CD44 antigen | P15379 |
| Leukosialin | P15702 |
| Glyceraldehyde-3-phosphate dehydrogenase | P16858 |
| Alpha-enolase | P17182 |
| Peptidyl-prolyl cis-trans isomerase A | P17742 |
| Triosephosphate isomerase | P17751 |
| Heat shock 70 kDa protein 1B | P17879 |
| Lysozyme C-1 | P17897 |
| Cathepsin D | P18242 |
| Cofilin-1 | P18760 |
| Keratin, type I cytoskeletal 19 | P19001 |
| Prothrombin | P19221 |
| Endoplasmic reticulum chaperone BiP | P20029 |
| Plasminogen | P20918 |
| Beta-enolase | P21550 |
| Embigin | P21995 |
| Integrin alpha-L | P24063 |
| Catalase | P24270 |
| Macrophage-capping protein | P24452 |
| Moesin | P26041 |
| Radixin | P26043 |
| Murinoglobulin-1 | P28665 |
| T-cell-specific surface glycoprotein CD28 | P31041 |
| Solute carrier family 2, facilitated glucose transporter member 3 | P32037 |
| Antithrombin-III | P32261 |
| Thrombospondin-1 | P35441 |
| Peroxiredoxin-1 | P35700 |
| Adenylyl cyclase-associated protein 1 | P40124 |
| Transketolase | P40142 |
| CD82 antigen | P40237 |
| Signal transducer and activator of transcription 1 | P42225 |
| F-actin-capping protein subunit beta | P47757 |
| Annexin A5 | P48036 |
| Proteasome subunit alpha type-2 | P49722 |
| Adenosylhomocysteinase | P50247 |
| Keratin, type II cytoskeletal 6A | P50446 |
| Myosin regulatory light chain 2, ventricular/cardiac muscle isoform | P51667 |
| Neutral amino acid transporter B | P51912 |
| Pyruvate kinase PKM | P52480 |
| Alpha-actinin-4 | P57780 |
| Elongation factor 2 | P58252 |
| Tropomyosin beta chain | P58774 |
| Actin, cytoplasmic 1 | P60710 |
| Eukaryotic initiation factor 4A-I | P60843 |
| Small ribosomal subunit protein eS8 | P62242 |
| 14-3-3 protein epsilon | P62259 |
| Small ribosomal subunit protein uS17 | P62281 |
| Histone H4 | P62806 |
| Guanine nucleotide-binding protein G/G/G subunit beta-2 | P62880 |
| Profilin-1 | P62962 |
| Ubiquitin-ribosomal protein eS31 fusion protein | P62983 |
| Heat shock cognate 71 kDa protein | P63017 |
| Eukaryotic translation initiation factor 5A-1 | P63242 |
| Actin, alpha skeletal muscle | P68134 |
| Tubulin beta-4B chain | P68372 |
| Pigment epithelium-derived factor | P97298 |
| Neuroplastin | P97300 |
| Plakophilin-1 | P97350 |
| Annexin A11 | P97384 |
| Tubulin beta-5 chain | P99024 |
| Proteasome subunit beta type-4 | P99026 |
| Retinol-binding protein 4 | Q00724 |
| Ubiquitin-like modifier-activating enzyme 1 | Q02053 |
| Junction plakoglobin | Q02257 |
| Protein-glutamine gamma-glutamyltransferase E | Q08189 |
| Keratin, type II cytoskeletal 2 epidermal | Q3TTY5 |
| Keratin, type II cytoskeletal 2 oral | Q3UV17 |
| Ubiquitin-associated and SH3 domain-containing protein A | Q3V3E1 |
| Cytoplasmic FMR1-interacting protein 2 | Q5SQX6 |
| Unconventional myosin-Ig | Q5SUA5 |
| Myosin-4 | Q5SX39 |
| Myosin-1 | Q5SX40 |
| Peroxiredoxin-2 | Q61171 |
| Plastin-2 | Q61233 |
| Keratin, type I cytoskeletal 15 | Q61414 |
| 5'-nucleotidase | Q61503 |
| Rab GDP dissociation inhibitor beta | Q61598 |
| Rho GDP-dissociation inhibitor 2 | Q61599 |
| Inter-alpha-trypsin inhibitor heavy chain H2 | Q61703 |
| Inter-alpha-trypsin inhibitor heavy chain H3 | Q61704 |
| Keratin, type I cytoskeletal 14 | Q61781 |
| Pregnancy zone protein | Q61838 |
| Transferrin receptor protein 1 | Q62351 |
| Vinculin | Q64727 |
| Clathrin heavy chain 1 | Q68FD5 |
| Alpha-2-macroglobulin-P | Q6GQT1 |
| Keratin, type I cytoskeletal 42 | Q6IFX2 |
| Keratin, type II cytoskeletal 1b | Q6IFZ6 |
| Transmembrane channel-like protein 8 | Q7TN58 |
| Desmoglein-1-beta | Q7TSF1 |
| Filamin-A | Q8BTM8 |
| Choline transporter-like protein 2 | Q8BY89 |
| Sodium-coupled neutral amino acid symporter 1 | Q8K2P7 |
| Vacuolar protein sorting-associated protein 37B | Q8R0J7 |
| Immunoglobulin superfamily member 8 | Q8R366 |
| Fibrinogen gamma chain | Q8VCM7 |
| Myosin-9 | Q8VDD5 |
| Sodium/potassium-transporting ATPase subunit alpha-1 | Q8VDN2 |
| Keratin, type II cytoskeletal 79 | Q8VED5 |
| Ras-related protein Rab-14 | Q91V41 |
| ATP-citrate synthase | Q91V92 |
| Myosin-7 | Q91Z83 |
| Serotransferrin | Q921I1 |
| Keratin, type II cytoskeletal 5 | Q922U2 |
| Disintegrin and metalloproteinase domain-containing protein 33 | Q923W9 |
| Rho GDP-dissociation inhibitor 1 | Q99PT1 |
| Golgi-associated plant pathogenesis-related protein 1 | Q9CYL5 |
| Charged multivesicular body protein 4b | Q9D8B3 |
| Elongation factor 1-gamma | Q9D8N0 |
| Inhibitor of carbonic anhydrase | Q9DBD0 |
| Keratin, type I cytoskeletal 17 | Q9QWL7 |
| Serine incorporator 3 | Q9QZI9 |
| Programmed cell death 6-interacting protein | Q9WU78 |
| EH domain-containing protein 1 | Q9WVK4 |
| Pantetheinase | Q9Z0K8 |
| Chloride intracellular channel protein 1 | Q9Z1Q5 |
| Thrombospondin-4 | Q9Z1T2 |
| Keratin, type I cytoskeletal 16 | Q9Z2K1 |
